# Substitution of acidic residues near the catalytic Glu131 leads to human HYAL1 activity at neutral pH via charge-charge interactions

**DOI:** 10.1371/journal.pone.0308370

**Published:** 2024-08-09

**Authors:** Tu Anh Nguyen, Trang Hoang, Thu-Thuy Nguyen, ChanSu Jeong, Trang Van Tran, Mal-Gi Choi, ChangWoo Lee

**Affiliations:** 1 Department of Biomedical Science and Center for Bio-Nanomaterials, Daegu University, Gyeongsan, South Korea; 2 Odysgen Inc., Daegu, South Korea; Roskilde Universitet, DENMARK

## Abstract

Human hyaluronidase 1 (HYAL1) and PH20 play vital roles in degrading hyaluronic acids through the substrate-assisted double displacement mechanism. While HYAL1, a lysosomal enzyme, functions optimally under acidic conditions, PH20, a sperm surface hyaluronidase, displays a broader pH range, from acidic to neutral. Our objective was to extend HYAL1’s pH range towards neutral pH by introducing repulsive charge-charge interactions involving the catalytic Glu131, increasing its p*K*_a_ as the proton donor. Substituting individual acidic residues in the β3-loop (S77D), β3′-β3″ hairpin (T86D and P87E), and at Ala132 (A132D and A132E) enabled HYAL1 to demonstrate enzyme activity at pH 7, with the mutants S77D, P87E, and A132E showing the highest activity in the substrate gel assay. However, double and triple substitutions, including S77D/T86D/A132E as found in the PH20 configuration, did not result in enhanced activity compared to single substitutions. Conversely, PH20 mutants with non-acidic substitutions, such as D94S in the β3-loop and D103T in the β3′-β3″ hairpin, significantly reduced activity within the pH range of 4 to 7. However, the PH20 mutant E149A, reciprocally substituted compared to A132E in HYAL1, exhibited activity similar to PH20 wild-type (WT) at pH 7. In a turbidimetric assay, HYAL1 mutants with single acidic substitutions exhibited activity similar to that of PH20 WT at pH 7. These results suggest that substituting acidic residues near Glu131 results in HYAL1 activity at neutral pH through electrostatic repulsion. This study highlights the significance of charge-charge interactions in both HYAL1 and PH20 in regulating the pH-dependent activity of hyaluronidases.

## Introduction

Hyaluronic acid (HA), a glycosaminoglycan and a major component of the extracellular matrix, consists of alternating dissacharides of β-D-(1→3) glucuronic acid (GlcA) and β-D-(1→4)-N-acetylglucosamine (GlcNAc) [1,2]. Vertebrate hyaluronidases (e.g., mammalian and venom hyaluronidases) (EC 3.2.1.35), classified under glycoside hydrolase family 56 in the carbohydrate-active enzyme database [3], target β-1,4 glycosidic linkages as endo-β-N-acetylhexosaminidases [4,5]. In humans, the hyaluronidase family comprise HYAL1 to HYAL4, and PH20 [6], sharing amino acid sequence identifies of 33–42% [5]. Among them, HYAL1, HYAL2, and PH20 function as active hyaluronidases [7–9], while HYAL3 is catalytically inactive [10,11], and HYAL4 has been reclassified as a chondroitin sulfate-specific enzyme, lacking HA hydrolysis activity [11].

Hyaluronidases catalyze the hydrolysis of HA through the substrate-assisted double displacement mechanism [12,13]. The active site typically contains two acidic residues, Asp–X–Glu (with X as Trp or Phe), where Glu serves as the catalytic residue [13,14]. The N-acetyl group of GlcNAc acts as the nucleophile, initiating glycosyl transfer by attacking the glycosidic bond [12,15]. The Glu residue serves dual roles in catalysis: as a general acid catalyst, it protonates the departing HA subunit’s hydroxyl group, and as a general base catalyst, it activates water molecules for glycosidic bond hydrolysis. Meanwhile, the Asp residue in the active site stabilizes the GlcNAc nitrogen transition state, supporting enzyme catalysis.

Although sharing the catalytic domain of a distorted (β/α)_8_-barrel fold and the same acidic residues in the active site [13,14], HYAL1 and PH20 exhibit distinct pH optima [4]. While HYAL1, primarily present in lysosomes and found in serum [16], displays a pH optimum of around 4 [4], PH20, located on the sperm surface and in the lysosome-derived acrosome [17], functions effectively across a wide pH range, from acidic to neutral [18]. The pH activity profiles of enzymes are intricately linked to the p*K*_a_ values of their catalytic residues, which are, in turn, influenced by the local environment and the specific amino acids present [19]. Several efforts have been made to extend HYAL1 activity to the neutral pH range. The introduction of a Gly-Ser-Gly-Ser tetrapeptide sequence in the substrate-binding loop (positions 207 to 221) of HYAL1 enhanced its activity at pH 5.9, expanding the binding cleft and improving HA polymer accessibility [20].

**Fig 1:**
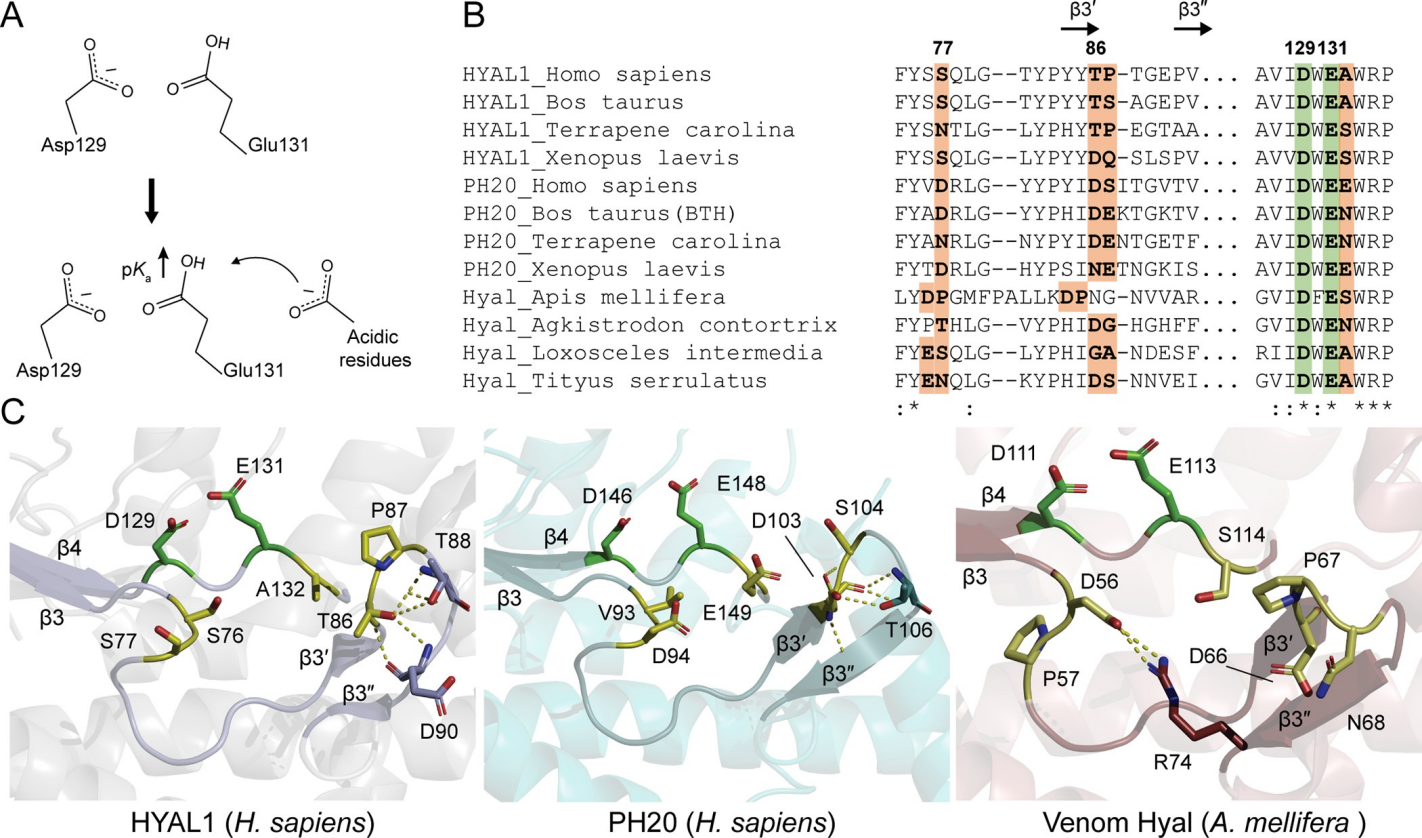
Charge-charge interactions involving the catalytic Glu131 in HYAL1 and sequence and structure comparison. (A) Schematic diagram highlighting induction of proximal negative charges by acidic residues to catalytic Glu131 in HYAL1, resulting in an increased p*K*_a_ as the proton donor. (B) Sequence comparison of HYAL1, PH20, and venom hyaluronidase orthologs. The HYAL1 sequences are from *Homo sapiens* (UniProt ID: Q12794), *Bos taurus* (UniProt ID: Q5E985), *Terrapene carolina* (UniProt ID: A0A674IY09), and *Xenopus laevis* (UniProt ID: A0A1L8GP88). The PH20 sequences are from *H*. *sapiens* (UniProt ID: P38567), *B*. *taurus* (UniProt ID: Q2YDK3), *T*. *carolina* (UniProt ID: A0A674IDS3), and *X*. *laevis* (UniProt ID: Q8UVT7). The venom hyaluronidase sequences are from *Loxosceles intermedia* (UniProt ID: R4J7Z9), *Apis mellifera* (UniProt ID: Q08169), *Agkistrodon contortrix* (NCBI ID: JAS04369.1), and *Tityus serrulatus* (NCBI ID: AHF72517.1). (C) Active-site structures of HYAL1, PH20, and bee venom hyaluronidase (*A*. *Mellifera*), depicting the proximity of the β3-loop and β3′-β3″ hairpin to the catalytic Glu residue. HYAL1 (PDB ID: 2PE4), PH20 (AlphaFold structure), and bee venom hyaluronidase (PDB ID: 1FCU).

Drawing from the influence of charge-charge interactions on the p*K*_a_ of enzyme ionizable groups through long-range and local electrostatic effects [21–23], our goal was to expand HYAL1’s pH range towards neutral pH. By introducing acidic residue substitutions near the catalytic residue Glu131, we aimed to elevate its p*K*_a_ as the proton donor via like-charge repulsion, facilitating HA hydrolysis at neutral pH (Fig 1A). Sequence and structural comparisons revealed shared acidic residues in the β3-loop and β3′-β3″ hairpin (referred to as the β-hairpin hereafter, unless specified otherwise) of PH20 and venom hyaluronidase orthologs, with variations observed in only one position where acidic residues are present (Fig 1B and 1C). Interestingly, certain PH20 variants, such as human PH20 and *Xenopus laevis* PH20 [24,25], feature a Glu residue adjacent to the catalytic Glu, while bovine testicular hyaluronidase (BTH) has Asn [26], and bee venom hyaluronidase has Ser at that particular position (Fig 1B) [27]. Additionally, bee venom hyaluronidase exhibits a salt bridge between Asp56, located in the β3-loop, and Arg74, situated in the β-hairpin (Fig 1C) [13]. A molecular dynamics simulation study on BTH showed significant flexibility in the β3-loop, as well as in the β4-loop, which contains the catalytic Glu [28].

In this study, we employed site-directed mutagenesis to introduce acidic residue substitutions near Glu131 in HYAL1 (β3-loop, β-hairpin, and Ala132). Single, double, and triple substitutions were introduced to assess the impact of increasing repulsive charge-charge interactions in HYAL1 involving Glu131. Additionally, non-acidic substitutions were performed in PH20 at positions corresponding to those in HYAL1 to explore the impact of charge-charge interactions in PH20. These modifications successfully extended HYAL1’s pH range from 4 to 7. However, the effects varied for PH20 depending on the positions of acidic residues at pH 7. These findings offer valuable insights into the pH-dependent behavior of hyaluronidases.

## Materials and methods

### Materials

The cDNAs of HYAL1 (hMU005315) and PH20 (hMU002604) were obtained from the Korea Human Gene Bank (Daejeon, South Korea). CHO-K1 cells were procured from the Korean Cell Line Bank (Seoul, South Korea). Both BTH (Cat. # H3506) and HA from *Streptococcus equi* (Cat. # 53747) were purchased from Sigma (St. Louis, MO, USA). Lipofectamine 3000 was purchased from ThermoFisher Scientific (Waltham, MA, USA), while restriction enzymes and Pfu polymerase were sourced from Enzynomics (Daejeon, South Korea). Other chemicals were acquired from Sigma unless otherwise stated.

### Construction of HYAL1 and PH20 expression plasmids

The cDNA encoding human HYAL1 was amplified from the pCMV-SPORT6-Hyal1 plasmid, introducing a XhoI site at the 5′-end and a 6x-His tag followed by a NotI site at the 3′ end via PCR with primers from S1 Table. The TA-*Hyal1* construct was digested with XhoI and NotI, and the excised *Hyal1* gene was integrated into the modified pSGHV0 vector [29]. This vector underwent removal of the hGH-His8-TEV recognition sequence via XhoI and NotI enzymes, resulting in the creation of the pSGHV1 vector in our laboratory.

The cDNA of human PH20 was PCR-amplified from the pBluescriptR-PH20 plasmid and subsequently subcloned into a TA vector. The forward primer added an additional XhoI site at the 5′ end, and the reverse primer incorporated NotI and a 6x-His tag at the 3′-end (S1 Table). The resulting TA-PH20 construct underwent XhoI and NotI digestion before being subcloned into the pSGHV1 vector. To eliminate the transmembrane domain of PH20, a truncation of the nucleotide sequence corresponding to amino acids 491 to 509 was performed, creating the pSGHV1-PH20-Δ491C vector. The signal sequence of PH20 was exchanged with that of HYAL1 using NEBuilder HiFi DNA Assembly (New England Biolabs, Ipswich, MA), with PH20-Δ491C serving as the template.

### Site-directed mutagenesis

Site-directed mutagenesis for single substitutions was conducted using nPfu polymerase to introduce mutations in the human *Hyal1* and *PH20* genes, guided by PCR primers detailed in S1 Table. For the *Hyal1* gene, we also established the following double and triple mutants: S77D/A132E and P87E/A132E in the A132E background, and S77D/T86D and T86D/P87E in the T86D background. Triple mutants, S77D/T86D/A132E and S77D/P87E/A132E, were achieved in the S77D/A132E background. For the *PH20* gene, the double mutants D94S/E149A and D103T/E149A were generated in the E149A background, while the triple mutant D94S/D103T/E149A was created using the D103T/E149A template. Following PCR amplification, the resulting products were subjected to treatment with DpnI at 37°C for 1 h before transformation into *E*. *coli* DH5α competent cells. We confirmed the accuracy of pSGHV1 mutant plasmids through DNA sequencing.

#### Structural analysis and multiple sequence alignment

For visualizing crystal structures, we used PyMOL (Schrodinger, LLC, New York, NY) for human HYAL1 (PDB ID: 2PE4) [14] and bee venom hyaluronidase (PDB ID: 1FCU) [13]. The AlphaFold structural models of human PH20 (UniProt ID: P38567) and other hyaluronidases with UniProt IDs were obtained from UniProt. In cases where predicted structures were unavailable, AlphaFold was employed to construct models [30], and the model quality was assessed through PROCHECK [31]. The Coulombic surface electrostatic potential of proteins was analyzed using ChimeraX without considering the initial pH [32]. The multiple sequence alignment of hyaluronidases was performed using Clustal Omega [33].

#### Expression of human HYAL1 and PH20 in CHO-K1 cells

CHO-K1 cells were grown up in T25 flasks in Dulbecco’s Modified Eagle Medium (DMEM) with 5% Fetal Bovine Serum (FBS) and 1% penicillin-streptomycin until reaching 90% confluency. Cells were treated with trypsin-EDTA and seeded to 12-well plates for the purpose of screening their activity. When the cell density reached 70–80%, plasmid transfection into the cells was performed using Lipofectamine 3000 following the manufacturer’s protocol. For the expression of HYAL1 WT and mutants, the transfection medium consisted of DMEM supplemented with 5% FBS and 1% penicillin-streptomycin. Conversely, when expressing PH20 WT and mutants, FBS and penicillin-streptomycin were excluded from the transfection medium to eliminate the effect of endogenous hyaluronidases present in the serum. After a 36-h incubation period, samples were collected for subsequent analysis, including total protein content determination using the Bradford assay and the hyaluronidase assay. The total protein content among WT and mutant samples was overall similar.

#### Substrate gel assay

The substrate gel assay for HA hydrolysis was conducted following the previously described method [34] with minor adjustments as follows. The culture supernatants collected from 12-well plates with CHO-K1 cells expressing hyaluronidases were loaded onto SDS gels (1.0 mg/mL HA for HYAL1 and 0.15 mg/mL HA for PH20) in equal volumes. Following SDS-PAGE, the gels were subsequently transferred to individual containers containing a 100 mM sodium chloride and 3% Triton X-100 buffer at pH 7.5. Renaturation was conducted at 4°C over a span of 2 h, with Triton X-100 solution replaced hourly. After renaturation, the gels were rinsed with distilled water to remove Triton X-100. Next, the gels were incubated with buffers of varying pH levels at 37°C for 14 h. The acetate buffer at pH 4 consisted of 100 mM sodium acetate, 100 mM sodium chloride, and glacial acetic acid. Phosphate buffers at pH 6 and 7 contained 100 mM sodium phosphate and 100 mM sodium chloride. Following incubation, the gels were immersed in an 1.0% Alcian Blue solution with 3% acetic acid for approximately 5 h. Finally, destaining was performed using a 10% acetic acid solution. Hyaluronidase activity appeared as clear bands against a blue background, and the band intensities were quantified using ImageJ [35]. These experiments were repeated three times, and the mean ± SD was presented.

#### Western blot

After SDS-PAGE as described in the substrate gel assay above, an extra SDS gel was retained for Western blot analysis. Proteins were transferred to a nitrocellulose membrane, followed by blocking with 5% non-fat dry milk in Tris-buffered saline containing 0.1% Tween-20 (TBST) for 1.5 h. Anti-His-tag monoclonal antibody (Santa Cruz Biotechnology, Santa Cruz, CA, USA) was applied to the membrane at a 1: 1000 ratio in TBST buffer containing 1% BSA. The membrane was then left overnight at 4°C. Following TBST washing, mouse IgGκ light chain binding protein conjugated to horseradish peroxidase (Santa Cruz Biotechnology) was used in TBST for 2 h at 25°C. After another TBST wash, target proteins were detected using enhanced chemiluminescence Western blotting substrate (ThermoFisher) and visualized through X-ray film exposure. The original and unadjusted images underlying all gel and blot results are shown in S1 Raw images.

#### Protein purification

The supernatant containing recombinant HYAL1 or PH20 proteins from CHO-K1 cells grown in 10-cm plates following the transient transfection as described above was collected by centrifugation and then adjusted to pH 8.0 by 2 M NaOH before loading into a 5-mL HisTrap column equilibrated with buffer A (50 mM Tris-HCl, pH 8.0, 50 mM NaCl, 5 mM imidazole, and 0.1 mM EDTA). The recombinant proteins were eluted with a linear gradient of 5–500 mM imidazole in buffer A on an AKTA go system (Cytiva, Marlborough, MA, USA). Proteins in elution fraction were loaded into a 1-mL Capto Q column. The proteins were subsequently eluted with a linear gradient of 50–1000 mM NaCl in buffer B (20 mM sodium phosphate, pH 7.4). All fractions containing the target proteins were pooled. For PH20 proteins, they were further purified by using size-exclusion chromatography on a Superdex 200 prep grade XK16 column in buffer B. The purified proteins were kept in buffer B with 5% glycerol and stored at −80°C for further experiments.

#### Turbidimetric assay

The hyaluronidase turbidimetric assay was conducted with minor modifications based on a previously described method [36]. Briefly, the enzyme was diluted in either acetate buffer (100 mM CH_3_COONa, 100 mM NaCl, pH 4) or phosphate buffer (100 mM sodium phosphate, 100 mM NaCl, pH 7). The mixture was equilibrated to 37°C for 10 min. Subsequently, a 0.03% (w/v) HA solution in either acetate buffer or phosphate buffer was added to the enzyme mixture and incubated at 37°C for 45 min. This was followed by the addition of 1 mL of acidic albumin solution (24 mM CH_3_COONa, 79 mM CH_3_COOH, and 0.1% (w/v) bovine serum albumin [BSA], pH 3.75), with a subsequent 10-min incubation at 25°C. The turbidity absorbance was measured at 600 nm using a Shimadzu UV-1800 spectrophotometer. Hyaluronidase activity was calculated as one unit causing a change in A_600_ of 0.330 per min at pH 5.35 at 37°C in a 1.2-mL reaction mixture. BTH (1000 units/mL) was used to generate standard curve. BTH was diluted in 20 mM sodium phosphate, 77 mM NaCl, and 0.01% BSA to achieve the desired final concentrations within a specific range (0.1 to 0.5), and enzyme activity was determined following the procedures described above.

#### Molecular dynamics (MD) and molecular docking simulations

The MD and molecular docking simulations were conducted at BioCode Ltd. (Liverpool, Merseyside, U.K.) using Maestro 12.0 (Schrödinger, LLC, New York, NY) for HYAL1 WT (PDB ID 2PE4) and A132E (AlphaFold structure model), with HA tetrasaccharide as a ligand obtained from bee venom hyaluronidase crystal structure (PDB ID: 1FCV). The OPLS4 force field [37] and SPC solvent model [38] were employed to define energetic parameters for all interactions. Protein preparation involved removing water molecules and optimizing structures using the protein preparation wizard. Ions were added as needed to maintain an electrically neutral state, and energy minimization was conducted for 1000 steps to resolve steric clashes or geometric issues. Solvent molecules were equilibrated with the fixed protein at 300 K, and the simulation ran for 100 ns in the NPT ensemble with a 2 fs time step. RMSD (root mean square deviation) calculations were based on C_α_-atoms across all trajectories up to 100 ns, while the RMSF (root mean square fluctuation) of C_α_-atoms at 300 K was estimated over equilibrated trajectories. Snapshots were generated every 100th frame throughout the simulation.

## Results

### Substituting acidic residues at Ala132 resulted in HYAL1 activity at neutral pH

**Fig 2:**
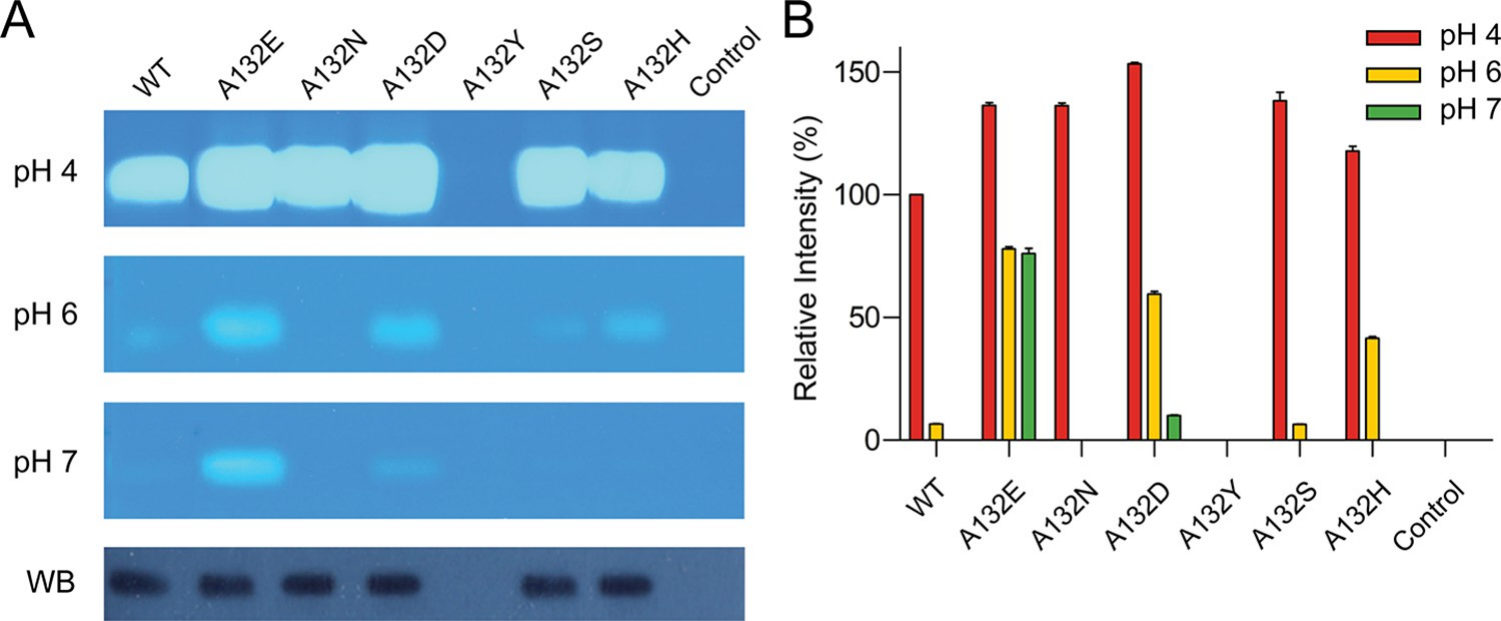
pH-dependent activity analysis of HYAL1 Ala132 mutants. (A) Substrate gel assay results at pH 4, 6, and 7 and Western blot analysis for HYAL1 WT and mutants. The blot was probed with an anti-His-tag monoclonal antibody. A representative set of experiments is shown. (B) Densitometric analysis of substrate gel assay. The activity of HYAL1 WT at pH 4 is set as 100%. Data presented are the means ± S.D. of three experiments.

Considering the proximity of Ala132 to the catalytic Glu131, we examined the effect of various Ala132 substitutions, including acidic (A132D, A132E), polar (A132N, A132S, A132Y), and basic (A132H) residues, on HYAL1 activity at elevated pH levels (Fig 2A and 2B). HYAL1 activity was evaluated using the substrate gel assay (1.0 mg/mL HA), with equal volumes of culture supernatant from transient transfection. All Ala132 mutants, except A132Y, exhibited an average 40% increase in activity at pH 4 compared to WT. Among the mutants, A132E, A132D, and A132H showed activity at pH 6, with approximately 78%, 60%, and 42% of WT activity at pH 4, respectively. Particuarly at pH 7, A132E showed the highest activity, reaching approximately 76% of WT activity at pH 4, whereas A132D exhibited approximately 10% of WT activity at this pH. However, A132H showed no activity at pH 7. Western blot analysis confirmed the expression of these mutants in CHO-K1 cells, except for A132Y, indicating that the lack of activity in A132Y is attributed to a failure in CHO-K1 cell expression (Fig 2A). These findings suggest that substituting Ala132 with Glu and Asp residues can extend HYAL1’s effective pH range to neutral pH. The enhanced activity of A132E compared to A132D can be attributed to the longer side chain of Glu compared to Asp, which facilitates a more effective charge interaction with Glu131. Furthermore, replacing Ala132 with larger side-chain residues is likely to destabilize the β4-loop, thereby enhancing activity at pH 4.

### Substituting acidic residues in the β3-loop and β-hairpin led to HYAL1 activity at neutral pH

**Fig 3:**
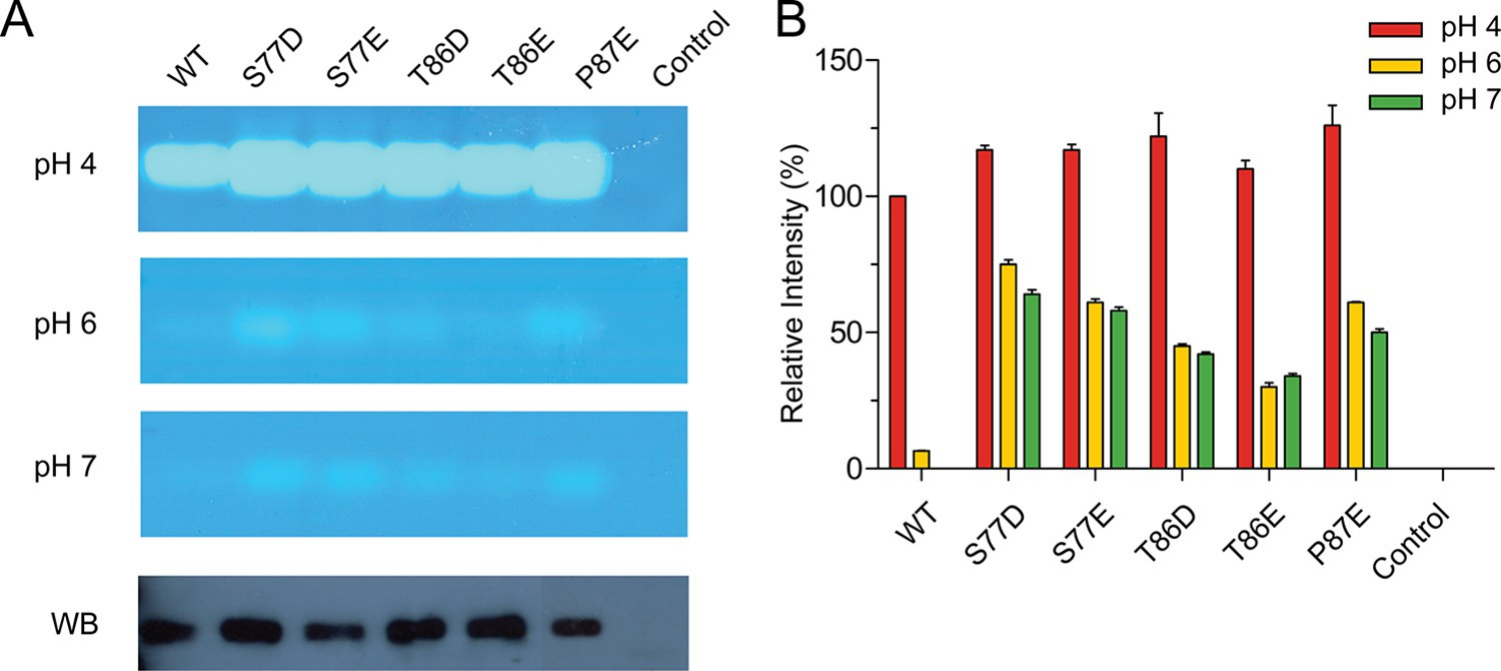
pH-dependent activity analysis of HYAL1 β3-loop and β-hairpin mutants. (A) Substrate gel assay results at pH 4, 6, and 7 and Western blot analysis for HYAL1 WT and mutants. The blot was probed with an anti-His-tag monoclonal antibody. A representative set of experiments is shown. (B) Densitometric analysis of substrate gel assay. The activity of HYAL1 WT at pH 4 is set as 100%. Data presented are the means ± S.D. of three experiments.

To evaluate the impact of acidic residues in the β3-loop and β-hairpin on HYAL1 activity at neutral pH, we introduced S77D and T86D substitutions, both involving Asp residues shared among PH20s and venom hyaluronidases (Fig 1B). To contrast the effects of Asp and Glu, we also generated S77E and T86E substitutions. We also examined the P87E substitution, situated at the apex of the β-hairpin loop, a feature frequently observed in PH20s from BTH, turtle, and *Xenopus laevis*, where Glu is prevalent at this position (Fig 1B). These mutants of the β3-loop and β-hairpin exhibited activity at pH 6 and 7 (Fig 3A and 3B). Specifically at pH 7, the S77D, S77E, and P87E mutants showed approximately 64%, 58%, and 50% of WT activity at pH 4, respectively, followed by the T86D and T86E mutants, which exhibited 42% and 34% of WT activity at pH 4, respectively. This reduced activity of the T86D and T86E mutants at pH 7 might be attributed to hydrogen bond formation between Asp86/Glu86 and the β3″-strand, as observed in the structural model of PH20, where Asp103 interacts with Thr106 (Fig 1C). Conversely, the P87E substitution facilitated HYAL1 activity, likely due to the favorable positioning of Glu at the apex of the β-hairpin loop. Furthermore, all these mutants exhibited approximately a 20% increase in activity compared to WT at pH 4. Western blot analysis confirmed the proper expression of these mutants in CHO-K1 cells (Fig 3A). Overall, these results demonstrate that acidic residues in the β3-loop and β-hairpin effectively provoke HYAL1 activity to neutral pH. Furthermore, these charge interactions may contribute to increased HYAL1 activity at pH 4, possibly through loop destabilization as observed in Ala132 mutants.

### Double and triple acidic substitutions did not yield enhanced HYAL1 activity compared to single substitutions at neutral pH

**Fig 4:**
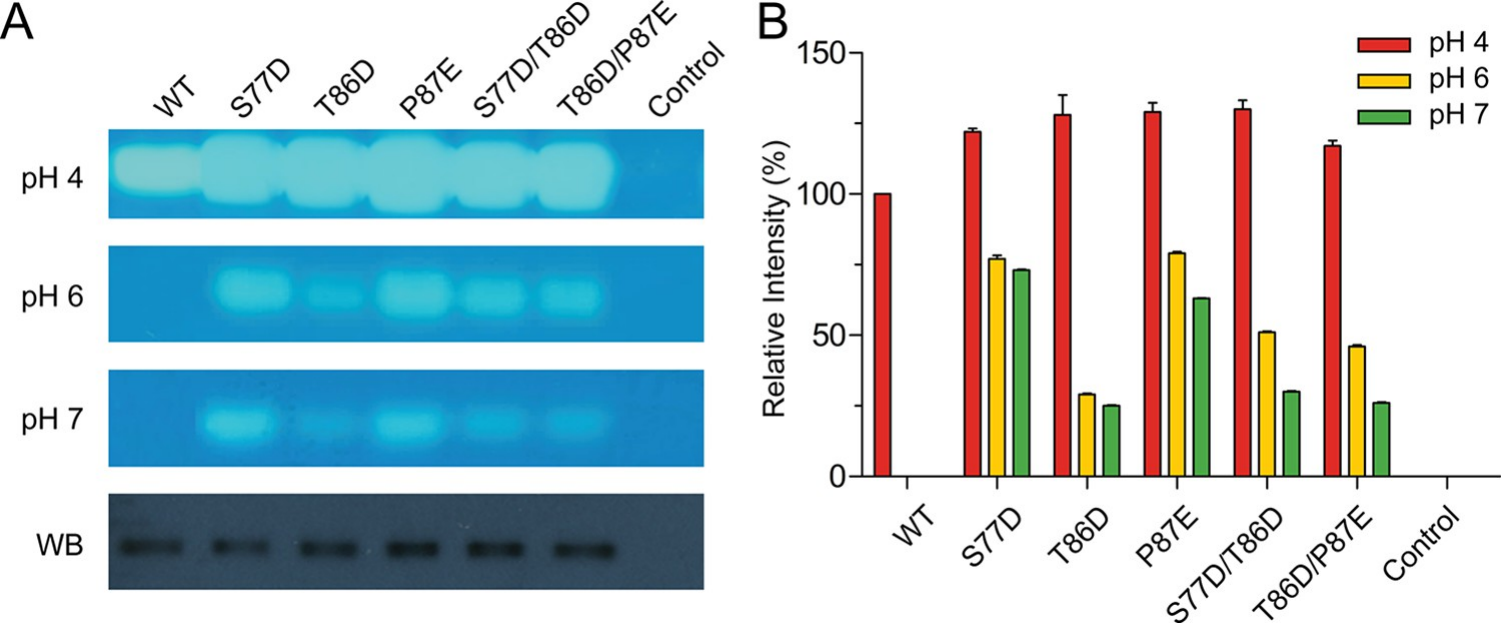
Characterization of HYAL1 β3-loop and β-hairpin mutants with double substitutions. (A) Substrate gel assay results at pH 4, 6, and 7 and Western blot analysis for HYAL1 WT and mutants. The blot was probed with an anti-His-tag monoclonal antibody. A representative set of experiments is shown. (B) Densitometric analysis of substrate gel assay. The activity of HYAL1 WT at pH 4 is set as 100%. Data presented are the means ± S.D. of three experiments.

We further examined the impact of double and triple acidic substitutions on HYAL1 activity at neutral pH. This involved introducing double acidic substitutions in both the β3-loop and β-hairpin (S77D/T86D and T86D/P87E), with the T86D/P87E substitution mirroring the configuration found in turtle PH20, which includes two acidic residues within the β-hairpin structure (Fig 1B). Similar to the single mutants, the double mutants (S77D/T86D and T86D/P87E) exhibited an approximately 30% and 17% increase in activity at pH 4 compared to WT, respectively (Fig 4A and 4B). However, at pH 6 and 7, these double mutants displayed intermediate activity levels compared to the respective single mutants, showing approximately 49% and 28% activity, respectively, relative to the WT activity at pH 4. The expression of these mutants was confirmed through Western blot analysis (Fig 4A). These findings indicate that, despite the increased potential for repulsive charge-charge interactions, there is no synergistic effect of substitutions in the β3-loop and β-hairpin on HYAL1 activity.

**Fig 5:**
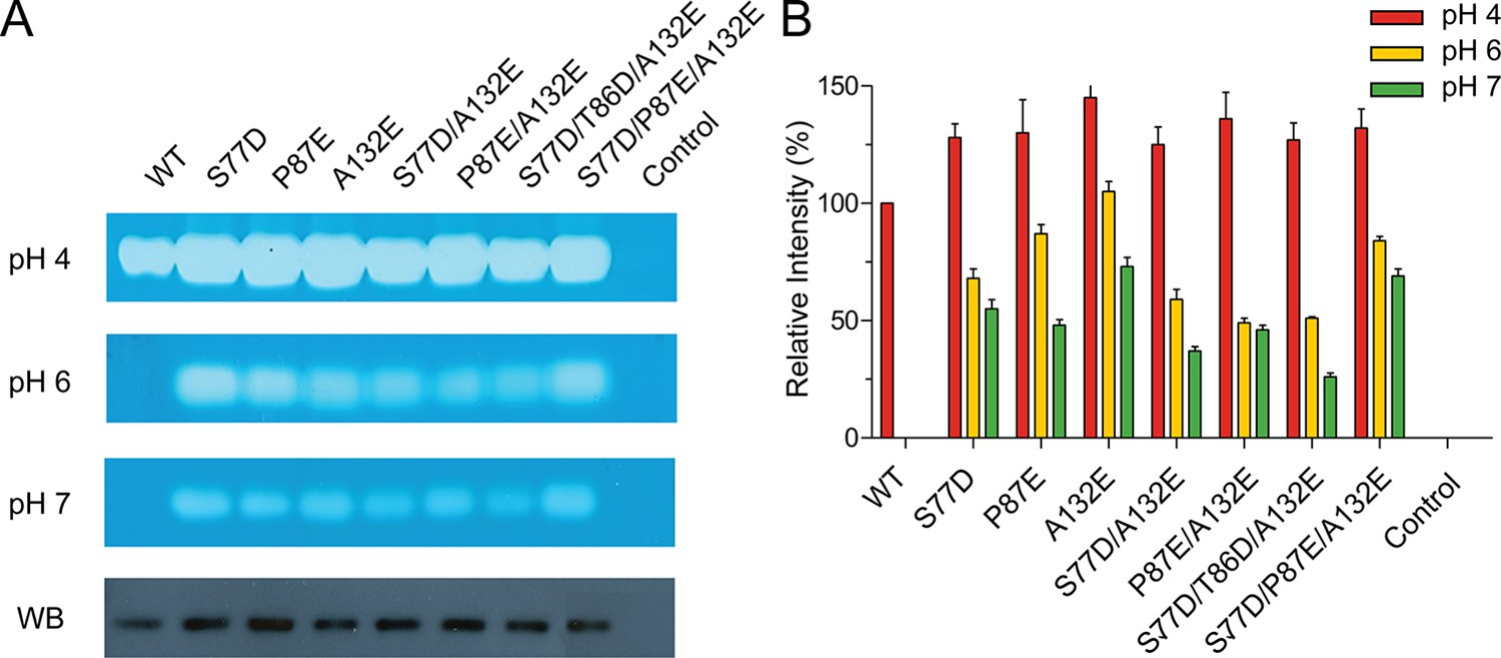
Effect of double and triple substitutions in the β3-loop, β-hairpin, and Ala132 on HYAL1 activity. (A) Substrate gel assay results at pH 4, 6, and 7 and Western blot analysis for HYAL1 WT and mutants. The blot was probed with an anti-His-tag monoclonal antibody. A representative set of experiments is shown. (B) Densitometric analysis of substrate gel assay. The activity of HYAL1 WT at pH 4 is set as 100%. Data presented are the means ± S.D. of three experiments.

We expanded our investigation to include the A132E substitution alongside substitutions in the β3-loop and β-hairpin, specifically S77D, T86D, and P87E. The double mutants (S77D/A132E and P87E/A132E) showed approximately 25% and 36% higher activity than WT at pH 4, respectively (Fig 5A and 5B). However, their activity at pH 6 and 7 was lower compared to the single mutants. As for the triple substitutions (S77D/T86D/A132E and S77D/P87E/A132E), their activity changes were influenced by T86D and P87E at both pH levels, with the latter showing higher activity than the former, which resembles the human PH20 configuration. Western blot analysis confirmed the expression of these mutants in CHO-K1 cells (Fig 5A). In summary, despite the increased repulsive charge-charge interactions, double and triple substitutions did not further enhance HYAL1 activity at neutral pH.

### Effect of non-acidic substitutions on enzyme activity in PH20

**Fig 6:**
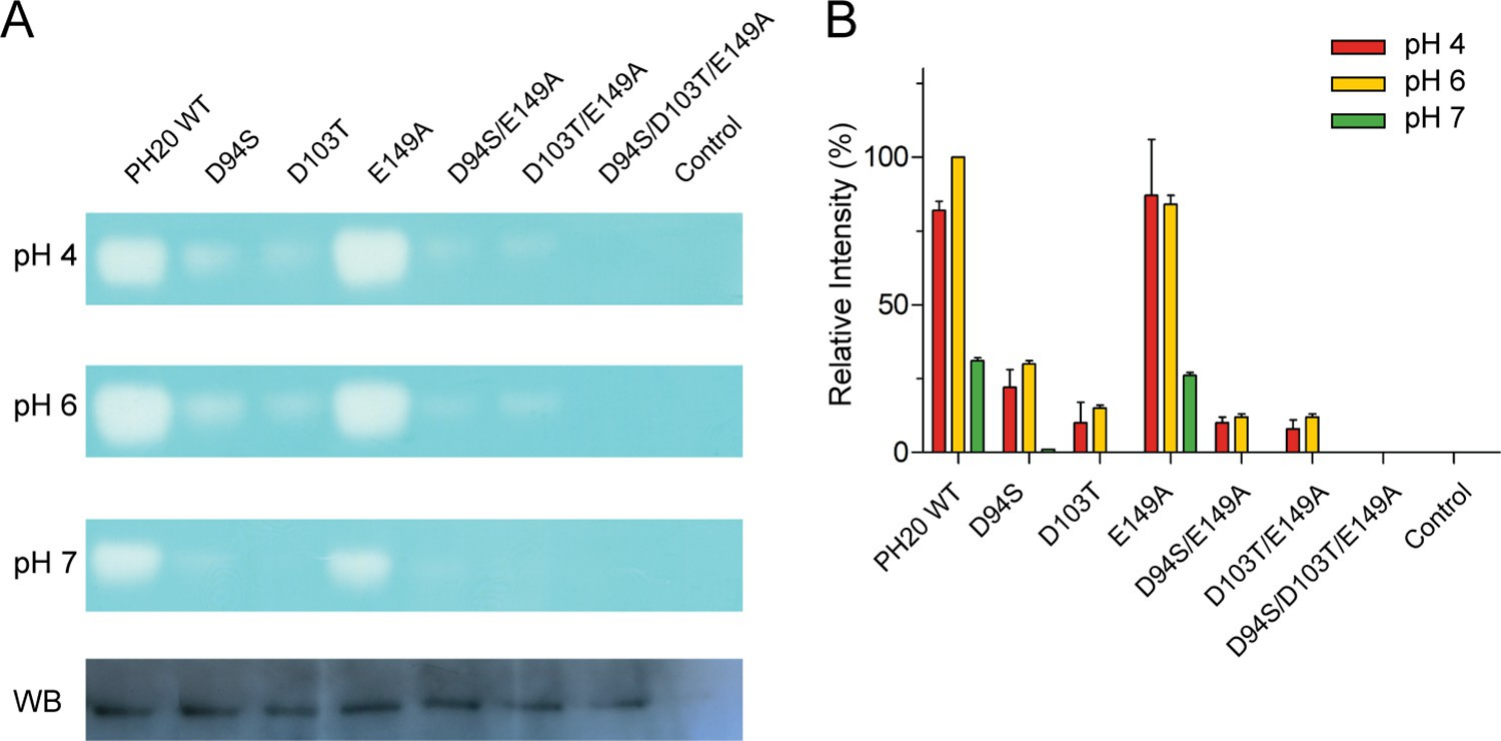
Effect of non-acidic substitutions in the β3-loop, β-hairpin, and Glu149 on PH20 activity. (A) Substrate gel assay results at pH 4, 6, and 7 and Western blot analysis for PH20 WT and mutants. The blot was probed with an anti-His-tag monoclonal antibody. A representative set of experiments is shown. (B) Densitometric analysis of substrate gel assay. The activity of PH20 WT at pH 6 is set as 100%. Data presented are the means ± S.D. of three experiments.

To validate our hypothesis on charge-charge interactions influencing HYAL1’s activity at neutral pH, we introduced reciprocal non-acidic substitutions in PH20 within the β3-loop, β-hairpin, and at Glu149 (D94S, D103T, and E149A), located close to catalytic Glu148. To address the lower enzyme activity due to reduced PH20 expression in CHO-K1 cells compared to HYAL1, we used a low concentration of HA (0.15 mg/mL) in SDS gel for Alcian Blue staining. PH20 WT exhibited its peak activity at pH 6 (100%), with approximately 82% activity at pH 4 and 31% at pH 7 (Fig 6A and 6B). As expected, non-acidic substitutions in the β3-loop (D94S) and β-hairpin (D103T) resulted in a complete loss of activity at pH 7. Intriguingly, the E149A mutant retained approximately 85% activity at pH 4–6 and 26% activity at pH 7 compared to the peak activity of PH20 WT at pH 6. However, mutants containing double substitutions (D94S/E149A and D103T/E149A) or triple substitutions (D94S/D103T/E149A) showed no detectable activity at pH 7, despite the presence of E149A. Western blot analysis confirmed the expression of PH20 mutants in CHO-K1 cells (Fig 6A). These results suggest that acidic residues both in the β3-loop (Asp94) and β-hairpin (Asp103) significantly influence the p*K*_a_ of Glu148 in PH20 for neutral pH activity. In contrast, Glu149 has an insignficant impact on neutral pH activity compared to the crucial role of Glu132 in HYAL1.

### Specific activity comparison of HYAL1 and PH20 WT and single mutants

**Fig 7:**
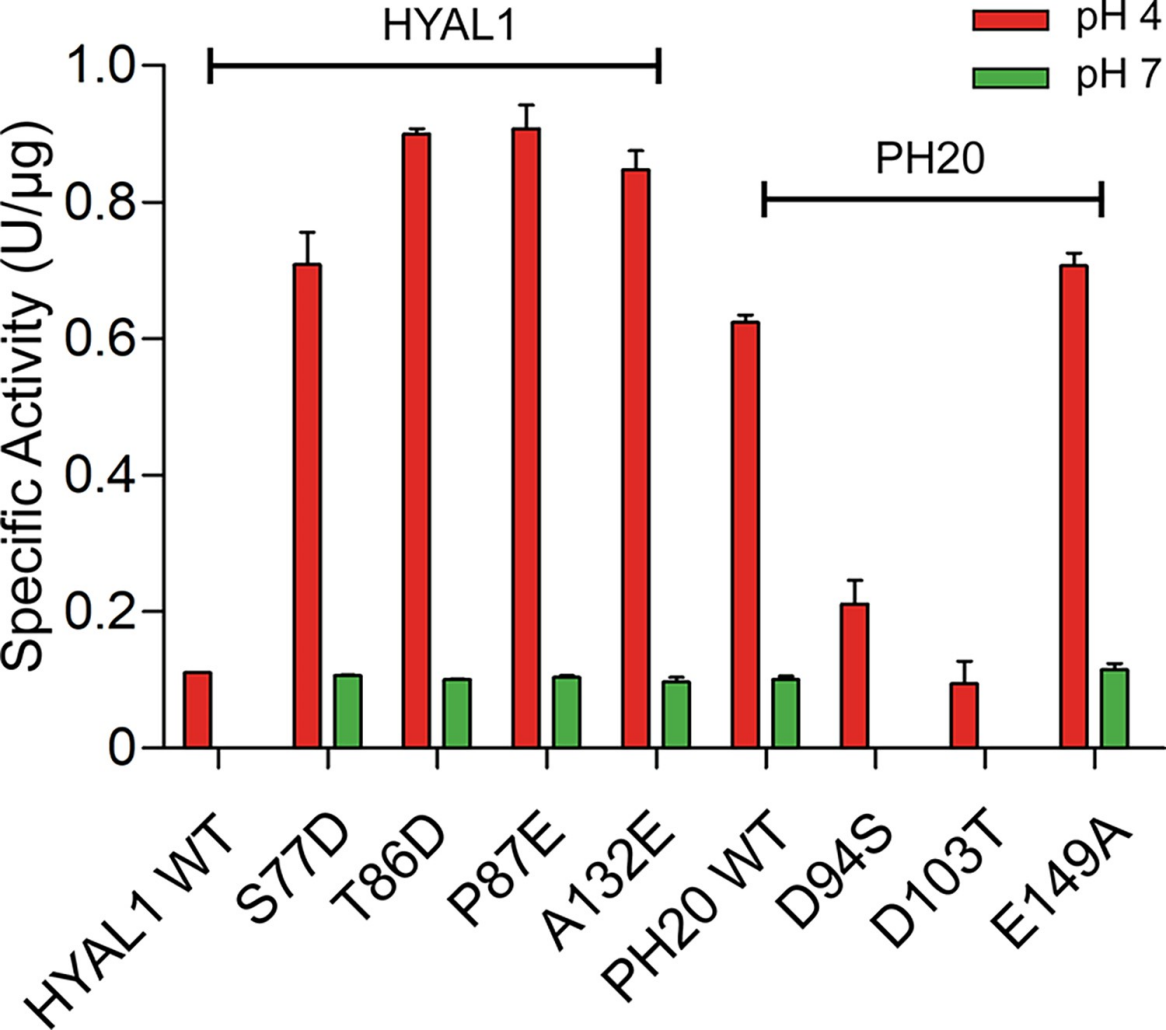
Specific activity of HYAL1 and PH20 WT and single mutants at pH 4 and 7. Enzyme activity was measured by incubating 0.03% (w/v) HA solution with enzyme mixture at 37°C for 45 min, followed by addition of acidic albumin solution at pH 3.75 and a 10-min incubation at 25°C. Turbidity absorbance was measured at 600 nm. Data presented are the means ± S.D. of three experiments.

HYAL1 and PH20 WT, along with their single mutants, were purified using nickel-chelate affinity chromatography and Capto Q anion-exchange chromatography, achieving over 85% purity (S1 Fig). Turbidimetric assay was utilized to assess their specific activity at pH 4 and 7. Unlike the substrate gel assay, which has limited HA amount in SDS-gel, resulting in an overall 20–40% higher activity in HYAL1 mutants compared to WT at pH 4 (Figs 2–6), HYAL1 single mutants exhibited 7–9-fold higher specific activity than WT at pH 4 (Fig 7). At pH 7, all HYAL1 single mutants showed similar activity to PH20 WT. Conversely, the non-acidic substitutions in PH20 (D94S and D103T) displayed 3- and 6-fold decreased activity at pH 4 compared to PH20 WT, respectively, with a complete loss of activity at pH 7. The specific activity of E149A was slightly higher than PH20 WT at both pH levels. These results are consistent with the enzyme activity shown in the substrate gel assay, highlighting that HYAL1 single mutants exhibit similar activity to PH20 WT at pH 7.

## Discussion

While many glycosidases commonly utilize a pair of acidic residues as catalytic residues, with one acting as a nucleophile and the other as a general acid-base catalyst, vertebrate hyaluronidases deviate due to their substrate-assisted double displacement mechanism [12,39,40]. These hyaluronidases feature Asp–X–Glu in the active site, where Glu acts as the catalytic residue, Asp plays a supporting role, and X (typically Trp or Phe) is positioned facing the opposite direction [13–15]. The close proximity of two negatively charged residues results in an elevated p*K*_a_ value and the protonation of one of them driven by electrostatic repulsion [13,21–23]. The intrinsic p*K*_a_ difference between Glu (intrinsic p*K*_a_ 4.3) and Asp (intrinsic p*K*_a_ 3.9) [41] supports Glu’s protonation and its role as the proton donor. The crystal structure of bee venom hyaluronidase shows a short hydrogen bond between Asp111 and Glu113 (with a distance of 3.8 Å), involving their four carboxyl oxygen atoms [13]. In HYAL1, Asp129 and Glu131 essentially share a proton at pH 4 [14].

This study demonstrates the crucial role of charge-charge interactions involving the catalytic Glu and nearby acidic residues in modulating the pH-dependent activity of both HYAL1 and PH20. The spatial arrangement of Asp129, Glu131, and nearby acidic residues in HYAL1 positions Glu131 between them, creating electrostatic repulsion that increases its p*K*_a_ and boosts activity at neutral pH. These results suggest that introducing a positively charged basic residue adjacent to a nearby negatively charged acidic residue to Glu131 may lower the acidic residue’s p*K*_a_ while increasing the basic residue’s p*K*_a_ through attractive charge-charge interactons [21–23]. These interactions could potentially elevate the p*K*_a_ of Glu131 due to enhanced electrostatic repulsion between Glu131 and the adjacent acidic residue. However, enhancing the protonation of Glu131 in HYAL1 through multiple acidic substitutions did not necessarily lead to increased enzymatic activity at neutral pH. On the other hand, HYAL1 A132H exhibited activity at pH 6, attributed to the β3-loop configuration influenced by a hydrogen bond between Ser76 and His132, impacting the charge interactions involving Glu131 (S4 Fig). Similarly, in the hyaluronidase from Cedar bark aphid (*Cinara cedri*), Asp116 in the β3-loop forms a salt bridge with His177, located adjacent to the catalytic Glu176 (S2 Fig).

We also examined additional acidic residue substitutions beyond the identified loop residues—S76D, Y85D, T88D, and G89D (S3 Fig). Interestingly, the Asp residue corresponding to the S76D substitution is conserved in specific venom hyaluronidases, including bee venom hyaluronidase (Fig 1B). Among these mutants, S76D, Y85D, and G89D showed no activity at pH 7, while T88D exhibited weak activity at this pH. The structural model of HYAL1 S76D showed a 3.4 Å hydrogen bond between Asp76 and Ser77, which hinders its activity at pH 7 (S4 Fig). This interaction is analogous to the Asp56-Arg74 salt bridge observed in bee venom hyaluronidase (Fig 1C), potentially influencing the charge interactions of Asp56 with the catalytic Glu113. However, the S77D mutant exhibits no hydrogen bond between Ser76 and Asp77 (S4 Fig), thus enabling its neutral pH activity through charge interactions with Glu131.

In contrast, the anomalous p*K*_a_ of the catalytic Glu148 in PH20 WT, caused by the nearby acidic residues, enables it to retain activity at pH 7. However, this characteristic does not prevent it from donating a proton to the hydroxyl leaving group of the HA subunit at pH 4; with a resulting approximately 20% decrease in activity at this pH (Fig 7). Substituting acidic-to-non-acidic residues in PH20 mutants is considered to decrease the p*K*_a_ of Glu148, leading to activity loss at pH 7. Our results indicate the importance of acidic residues (Asp94 and Asp103) within the flexible β3-loop for PH20 activity [28]. On the contrary, the impact of Glu149 on PH20 activity was minor, as evidenced by the retention of activity by the E149A mutant at pH 7. These results further underscore the distinct active-site conformations between HYAL1 and PH20.

Additionally, these results also emphasize that at least a single acidic residue near Glu131 is sufficient for HYAL1 to achieve neutral pH activity compared to PH20 possessing three acidic residues adjacent to Glu148. However, certain PH20 variants, such as those found in turtles, feature only acidic residues in the β-hairpin (Asp-Glu), showing a preference for either the β3-loop or the β-hairpin positions (Fig 1B). This pattern is also observed in venom hyaluronidases, which often have acidic residues in either the β3-loop or the β-hairpin (Fig 1B). These findings support the versatility of venom hyaluronidases across broad pH ranges. For instance, bee venom hyaluronidases show activity over a pH range of 3–8 (with a peak at 3.8) [42], while snake venom hyaluronidases exhibit optimal activity between pH 5–6 [43], enabling them to function effectively as spreading factors. While initially considered distant from the active site in bee venom hyaluronidase [13], our findings propose that the hydrophilic loop 65–71 (KDPNGNV) joining the β-hairpin may engage in charge-charge interactions with Glu113 (Fig 1C). The sequence comparison suggests that PH20s employ a sophisticated strategy to adjust the working pH range through multiple acidic residues, while venom hyaluronidases often rely on acidic residues in either the β3-loop or β-hairpin (S5 Fig).

MD and molecular docking simulations were conducted to assess changes in the ligand binding site of HYAL1 WT and A132E using an HA tetrasaccharide as a ligand. Unexpectedly, the HA tetrasaccharides showed an affinity for sites in each protein’s N- and C-terminal regions (S6 Fig), deviating from the expected binding groove observed in bee venom hyaluronidase [13]. RMSD values, which measure the average distance between superimposed atomic structures over time, and RMSF values, which quantify the flexibility of C_α_ atoms during the simulation, were extracted from the MD trajectory for both WT and A132E (S7 Fig). Equilibrium was reached within 20 ns for both proteins. The ligand binding to WT remained unstable throughout the simulation, while stable binding was observed in A132E after 40 ns. Both WT and A132E exhibited similar binding affinities of −9.6 and −9.4 kcal/mol, respectively. Interestingly, despite similar RMSF values in both structures, A132E demonstrated a significant increase in residues binding to the ligand (S6 Fig), particularly within the ligand-binding pocket (S7 Fig). This observation suggests that Glu132 influences the positioning of the ligand towards the binding pocket in the A132E structure, in addition to its role in β4-loop flexibility and charge interactions.

The net charge of the protein surface is also crucial for the activity of HYAL1 [20,44]. We examined the structures of HYAL1, PH20, and venom hyaluronidase orthologs to assess surface electrostatic potential. HYAL1’s active site displayed predominantly negative charges at Asp129 and Glu131, while PH20s and venom hyaluronidases showed enhanced negative charges in the nearby β3-loop and β-hairpin, alongside corresponding Asp and Glu residues (S8 Fig). The increased activity of HYAL1 mutants at pH 4 could be attributed to altered electrostatic potential affecting substrate binding. In contrast, the abundance of negative charges in the substrate binding region of PH20 had a comparatively lesser effect on enzyme activity.

In conclusion, both HYAL1 and PH20 utilize charge-charge interactions with the catalytic Glu to achieve their optimal pH conditions, albeit differently. Our study suggests that neutral pH-active HYAL1, facilitated by acidic residue substitutions, presents a versatile alternative for PH20 for various clinical applications, including subcutaneous delivery of therapeutic antibodies, tumor HA degradation for cancer therapy, enhancement of local anesthetic spread and absorption during ophthalmological surgery, and treatment for HA filler complications [45–48]. Comprehensive understanding of the pH-dependent mechanisms of hyaluronidases will expand their therapeutic potential, necessitating further investigation to elucidate the intricate interplay between charge-charge interactions and active-site conformations.

## Supporting information

S1 Table List of primers for cloning and site-directed mutagenesis. (PDF)

S1 Fig SDS-PAGE gel after protein purification. (PDF)

S2 Fig Structural models of HYAL1 A132H (A) and Cedar bark aphid hyaluronidase (*Cinara cedri*) (B).
(PDF)

S3 Fig pH-dependent activity of HYAL1 extended β-hairpin region mutants (A) and Ser76 mutants (B).
(PDF)

S4 Fig Structural models of HYAL1 S76D (A) and S77D (B).
(PDF)

S5 Fig Sequence comparison of hyaluronidase orthologs. (PDF)

S6 Fig Docking simulation of HYAL1 WT and A132E with an HA tetrasaccharide. (PDF)

S7 Fig Molecular dynamics analysis of HYAL1 WT and A132E with an HA tetrasaccharide. (PDF)

S8 Fig Surface electrostatic potential of hyaluronidases. (PDF)

S1 Raw images The original and unadjusted images underlying all blot and gel results. (PDF)

## References

[1] Simpson M, Schaefer L, Hascall V, Esko JD. Hyaluronan. In: Varki A, Cummings RD, Esko JD, Stanley P, Hart GW, Aebi M, et al., editors. Essentials of glycobiology. 4th ed. Cold Spring Harbor (NY): Cold Spring Harbor Laboratory Press; 2022. pp. 205–16.

[2] Kobayashi T, Chanmee T, Itano N. Hyaluronan: metabolism and function. Biomolecules. 2020;10(11):1525. doi: 10.3390/biom10111525 . 33171800 PMC7695009

[3] Drula E, Garron ML, Dogan S, Lombard V, Henrissat B, Terrapon N. The carbohydrate-active enzyme database: functions and literature. Nucleic Acids Res. 2022;50(D1):D571–D577. doi: 10.1093/nar/gkab1045 . 34850161 PMC8728194

[4] El-Safory NS, Fazary AE, Lee C-K. Hyaluronidases, a group of glycosidases: Current and future perspectives. Carbohydr Polym. 2010;81(2):165–81. 10.1016/j.carbpol.2010.02.047.

[5] Stern R, Jedrzejas MJ. Hyaluronidases: their genomics, structures, and mechanisms of action. Chem Rev. 2006;106(3):818–39. doi: 10.1021/cr050247k . 16522010 PMC2547145

[6] Csoka AB, Frost GI, Stern R. The six hyaluronidase-like genes in the human and mouse genomes. Matrix Biol. 2001;20(8):499–508. doi: 10.1016/s0945-053x(01)00172-x . 11731267

[7] Afify AM, Stern M, Guntenhoner M, Stern R. Purification and characterization of human serum hyaluronidase. Arch Biochem Biophys. 1993;305(2):434–41. doi: 10.1006/abbi.1993.1443 . 8373180

[8] Lepperdinger G, Strobl B, Kreil G. HYAL2, a human gene expressed in many cells, encodes a lysosomal hyaluronidase with a novel type of specificity. J Biol Chem. 1998;273(35):22466–70. doi: 10.1074/jbc.273.35.22466 . 9712871

[9] Gmachl M, Sagan S, Ketter S, Kreil G. The human sperm protein PH-20 has hyaluronidase activity. FEBS Lett. 1993;336(3):545–8. doi: 10.1016/0014-5793(93)80873-s . 8282124

[10] Harada H, Takahashi M. CD44-dependent intracellular and extracellular catabolism of hyaluronic acid by hyaluronidase-1 and -2. J Biol Chem. 2007;282(8):5597–607. doi: 10.1074/jbc.M608358200 . 17170110

[11] Atmuri V, Martin DC, Hemming R, Gutsol A, Byers S, Sahebjam S, et al. Hyaluronidase 3 (HYAL3) knockout mice do not display evidence of hyaluronan accumulation. Matrix Biol. 2008;27(8):653–60. https://10.1016/j.matbio.2008.07.006. doi: 10.1016/j.matbio.2008.07.006 . 18762256

[12] Jedrzejas MJ, Stern R. Structures of vertebrate hyaluronidases and their unique enzymatic mechanism of hydrolysis. Proteins. 2005;61(2):227–38. https://10.1002/prot.20592. doi: 10.1002/prot.20592 . 16104017

[13] Markovic-Housley Z, Miglierini G, Soldatova L, Rizkallah PJ, Muller U, Schirmer T. Crystal structure of hyaluronidase, a major allergen of bee venom. Structure. 2000;8(10):1025–35. doi: 10.1016/s0969-2126(00)00511-6 . 11080624

[14] Chao KL, Muthukumar L, Herzberg O. Structure of human hyaluronidase-1, a hyaluronan hydrolyzing enzyme involved in tumor growth and angiogenesis. Biochemistry. 2007;46(23):6911–20. doi: 10.1021/bi700382g . 17503783

[15] Zhang L, Bharadwaj AG, Casper A, Barkley J, Barycki JJ, Simpson MA. Hyaluronidase activity of human Hyal1 requires active site acidic and tyrosine residues. J Biol Chem. 2009;284(14):9433–42. doi: 10.1074/jbc.M900210200 . 19201751 PMC2666596

[16] Frost GI, Csoka AB, Wong T, Stern R. Purification, cloning, and expression of human plasma hyaluronidase. Biochem Biophys Res Commun. 1997;236(1):10–5. doi: 10.1006/bbrc.1997.6773 . 9223416

[17] Cherr GN, Yudin AI, Overstreet JW. The dual functions of GPI-anchored PH-20: hyaluronidase and intracellular signaling. Matrix Biol. 2001;20(8):515–25. doi: 10.1016/s0945-053x(01)00171-8 . 11731269

[18] Martin-Deleon PA. Germ-cell hyaluronidases: their roles in sperm function. Int J Androl. 2011;34(5 Pt 2):e306–18. doi: 10.1111/j.1365-2605.2010.01138.x . 21418239

[19] Joshi MD, Sidhu G, Nielsen JE, Brayer GD, Withers SG, McIntosh LP. Dissecting the electrostatic interactions and pH-dependent activity of a family 11 glycosidase. Biochemistry. 2001;40(34):10115–39. https://10.1021/bi0105429. . 11513590

[20] Reitinger S, Mullegger J, Greiderer B, Nielsen JE, Lepperdinger G. Designed human serum hyaluronidase 1 variant, HYAL1DeltaL, exhibits activity up to pH 5.9. J Biol Chem. 2009;284(29):19173–7. doi: 10.1074/jbc.C109.004358 . 19478093 PMC2740539

[21] Harris TK, Turner GJ. Structural basis of perturbed p*K*_a_ values of catalytic groups in enzyme active sites. IUBMB Life. 2002;53(2):85–98. doi: 10.1080/15216540211468 . 12049200

[22] Pace CN, Grimsley GR, Scholtz JM. Protein ionizable groups: p*K* values and their contribution to protein stability and solubility. J Biol Chem. 2009;284(20):13285–9. doi: 10.1074/jbc.R800080200 . 19164280 PMC2679426

[23] Li SF, Cheng F, Wang YJ, Zheng YG. Strategies for tailoring pH performances of glycoside hydrolases. Crit Rev Biotechnol. 2023;43(1):121–41. doi: 10.1080/07388551.2021.2004084 . 34865578

[24] Lin Y, Kimmel LH, Myles DG, Primakoff P. Molecular cloning of the human and monkey sperm surface protein PH-20. Proc Natl Acad Sci U S A. 1993;90(21):10071–5. doi: 10.1073/pnas.90.21.10071 . 8234258 PMC47715

[25] Reitinger S, Mullegger J, Lepperdinger G. *Xenopus* kidney hyaluronidase-1 (XKH1), a novel type of membrane-bound hyaluronidase solely degrades hyaluronan at neutral pH. FEBS Lett. 2001;505(2):213–6. doi: 10.1016/s0014-5793(01)02813-7 . 11566178

[26] Meyer MF, Kreil G, Aschauer H. The soluble hyaluronidase from bull testes is a fragment of the membrane-bound PH-20 enzyme. FEBS Lett. 1997;413(2):385–8. doi: 10.1016/s0014-5793(97)00936-8 . 9280317

[27] Gmachl M, Kreil G. Bee venom hyaluronidase is homologous to a membrane protein of mammalian sperm. Proc Natl Acad Sci U S A. 1993;90(8):3569–73. doi: 10.1073/pnas.90.8.3569 . 7682712 PMC46342

[28] Amin M, Barzegari E, Pourshohod A, Zeinali M, Jamalan M. 3D structure prediction, dynamic investigation and rational construction of an epitope-masked thermostable bovine hyaluronidase. Int J Biol Macromol. 2021;187:544–53. doi: 10.1016/j.ijbiomac.2021.07.098 . 34298049

[29] Leahy DJ, Dann CE 3rd, Longo P, Perman B, Ramyar KX. A mammalian expression vector for expression and purification of secreted proteins for structural studies. Protein Expr Purif. 2000;20(3):500–6. doi: 10.1006/prep.2000.1331 . 11087690

[30] Jumper J, Evans R, Pritzel A, Green T, Figurnov M, Ronneberger O, et al. Highly accurate protein structure prediction with AlphaFold. Nature. 2021;596(7873):583–9. doi: 10.1038/s41586-021-03819-2 . 34265844 PMC8371605

[31] Laskowski RA, Rullmannn JA, MacArthur MW, Kaptein R, Thornton JM. AQUA and PROCHECK-NMR: programs for checking the quality of protein structures solved by NMR. J Biomol NMR. 1996;8(4):477–86. doi: 10.1007/BF00228148 . 9008363

[32] Goddard TD, Huang CC, Meng EC, Pettersen EF, Couch GS, Morris JH, et al. UCSF ChimeraX: Meeting modern challenges in visualization and analysis. Protein Sci. 2018;27(1):14–25. doi: 10.1002/pro.3235 . 28710774 PMC5734306

[33] Sievers F, Higgins DG. Clustal Omega for making accurate alignments of many protein sequences. Protein Sci. 2018;27(1):135–45. doi: 10.1002/pro.3290 . 28884485 PMC5734385

[34] Guntenhoner MW, Pogrel MA, Stern R. A substrate-gel assay for hyaluronidase activity. Matrix. 1992;12(5):388–96. doi: 10.1016/s0934-8832(11)80035-1 . 1484506

[35] Schneider CA, Rasband WS, Eliceiri KW. NIH Image to ImageJ: 25 years of image analysis. Nat Methods. 2012;9(7):671–5. doi: 10.1038/nmeth.2089 . 22930834 PMC5554542

[36] Tolksdorf S, Mc CM, et al. The turbidimetric assay of hyaluronidase. J Lab Clin Med. 1949;34(1):74–89. . 18106411

[37] Lu C, Wu C, Ghoreishi D, Chen W, Wang L, Damm W, et al. OPLS4: improving force field accuracy on challenging regimes of chemical space. J Chem Theory Comput. 2021;17(7):4291–300. doi: 10.1021/acs.jctc.1c00302 . 34096718

[38] Frank HS, Wen W-Y. Ion-solvent interaction. Structural aspects of ion-solvent interaction in aqueous solutions: a suggested picture of water structure. Disc Faraday Soc. 1957;24(0):133–40. https://10.1039/DF9572400133.

[39] Vuong TV, Wilson DB. Glycoside hydrolases: catalytic base/nucleophile diversity. Biotechnol Bioeng. 2010;107(2):195–205. doi: 10.1002/bit.22838 . 20552664

[40] Vasella A, Davies GJ, Bohm M. Glycosidase mechanisms. Curr Opin Chem Biol. 2002;6(5):619–29. doi: 10.1016/s1367-5931(02)00380-0 . 12413546

[41] Grimsley GR, Scholtz JM, Pace CN. A summary of the measured p*K* values of the ionizable groups in folded proteins. Protein Sci. 2009;18(1):247–51. doi: 10.1002/pro.19 . 19177368 PMC2708032

[42] Reitinger S, Boroviak T, Laschober GT, Fehrer C, Mullegger J, Lindner H, et al. High-yield recombinant expression of the extremophile enzyme, bee hyaluronidase in *Pichia pastoris*. Protein Expr Purif. 2008;57(2):226–33. doi: 10.1016/j.pep.2007.10.001 . 18024155

[43] Kemparaju K, Girish KS, Nagaraju S. Hyaluronidases, a neglected class of glycosidases from snake venom: beyond a spreading factor. In: Mackessy SP, editor. Handbook of venoms and toxins of reptiles. Boca Raton (FL): CRC Press; 2010. pp. 237–58.

[44] Lenormand H, Deschrevel B, Vincent JC. pH effects on the hyaluronan hydrolysis catalysed by hyaluronidase in the presence of proteins: Part I. Dual aspect of the pH-dependence. Matrix Biol. 2010;29(4):330–7. doi: 10.1016/j.matbio.2009.12.007 . 20043995

[45] Locke KW, Maneval DC, LaBarre MJ. ENHANZE drug delivery technology: a novel approach to subcutaneous administration using recombinant human hyaluronidase PH20. Drug Deliv. 2019;26(1):98–106. doi: 10.1080/10717544.2018.1551442 . 30744432 PMC6394283

[46] Thompson CB, Shepard HM, O’Connor PM, Kadhim S, Jiang P, Osgood RJ, et al. Enzymatic depletion of tumor hyaluronan induces antitumor responses in preclinical animal models. Mol Cancer Ther. 2010;9(11):3052–64. doi: 10.1158/1535-7163.MCT-10-0470 . 20978165

[47] Weber GC, Buhren BA, Schrumpf H, Wohlrab J, Gerber PA. Clinical applications of hyaluronidase. In: Labrou N, editor. Therapeutic enzymes: function and clinical implications. Singapore: Springer Singapore; 2019. pp. 255–77. 10.1007/978-981-13-7709-9_12 31482503

[48] Cavallini M, Gazzola R, Metalla M, Vaienti L. The role of hyaluronidase in the treatment of complications from hyaluronic acid dermal fillers. Aesthet Surg J. 2013;33(8):1167–74. doi: 10.1177/1090820X13511970 . 24197934

