# Supplementary material for: Substitution of acidic residues near the catalytic Glu131 leads to human HYAL1 activity at neutral pH via charge-charge interactions
Source: PLoS One. 2024 Aug 9;19(8):e0308370. doi: 10.1371/journal.pone.0308370 (PMC11315327; doi:10.1371/journal.pone.0308370)
Supplement: S1 Table — (PDF) [file pone.0308370.s001.pdf]

Table S1. List of primers for cloning and site-directed mutagenesis.

		Primer sequence
HYAL1 WT	Forward	5′-ctcgaggccaccatggcagcccacctgcttcc-3′ (XhoI site underlined)
	Reverse	5′-gcggccgctcagtggtgatggtgatgatgccacatgc-3′ (NotI site underlined)
S76D	Forward	5′ -gacaattttctatGACtcccag-3′
	Reverse	5′ -ccagctgggaGTCataga-3′
S77D	Forward	5′ -caattttctatagcGACcagct-3′
	Reverse	5’-cccagctgCTCgctat-3’
S77E	Forward	5′ -ctatagcGAGcagctggg-3′
	Reverse	5′ -cccagctgCTCgctat-3′
Y85D	Forward	5′ -acctacccctacGACacgccc-3′
	Reverse	5′ -agtgggcgtGTCgtaggggta-3′
T86D	Forward	5′ -ctacccctactacGATcccac-3′
	Reverse	5′ -ccccagtgggATCgtagtag-3′
T86E	Forward	5′ -ctacccctactacGAGcccac-3′
	Reverse	5′ -ccccagtgggCTCgtagtag-3′
P87D	Forward	5′ -ccctactacacgGACactgg-3′
	Reverse	5′ -ctccccagtGTCcgtgtag-3′
T86D/P87E	Forward	5′ -ccctactacGATGAAactgg-3′

	Reverse	5′ -ccagtTTCATCgtagtaggg-3′
T88D	Forward	5′ -tactacacgcccGATggggagc-3′
	Reverse	5′ -aggctccccATCgggcgt-3′
G89D	Forward	5′ -tacacgcccactGATgagcc-3′
	Reverse	5′ -cacaggctcATCagtgggc-3′
A132N	Forward	5′ -tgggagAACtggcgcccac-3′
	Reverse	5′ -gcgtgggcgccaGTTctc-3′
A132D	Forward	5′ -catcgactgggagGATtggcg-3′
	Reverse	5′ -cgccaATCctcccagtcgatg-3′
A132E	Forward	5′ -tgggagGAAtggcgcccac-3′
	Reverse	5′ -cagcgtgggcgccaTTCctc-3′
A132S	Forward	5’-catcgactgggagAGTtggcg-3’
	Reverse	5’-cgccaACTctcccagtcgatg-3’
A132H	Forward	5’-catcgactgggagCATtggcg-3’
	Reverse	5’-cgccaATGctcccagtcgatg-3’
A132Y	Forward	5′ -catcgactgggagTATtggcgc-3′
	Reverse	5′ -gcgccaATActcccagtcgatg-3′
PH20 WT	Forward	5′-ctcgagatgggagtgctaaaattcaagcacatctttttcag-3′ (XhoI site underlined)
	Reverse	5′-gcggccgcttaatgatgatgatgatgatggaagaaaccaattctg-3′ (NotI site underlined)

PH20 ΔC491	Forward	5′-catcatcatcatcatcattaagc-3′
	Reverse	5′-caccctccacactatctcatcatc-3′
PH20 with the HYAL1 signal sequence	Forward	5′- aagcttggctgtggaatgtg -3′ (HYAL1 template)
	Reverse	5′- gccttgggccatatcgagtaag -3′ (HYAL1 template)
	Forward	5′- gatatggcccaaggcctgaatttcagagcacctc -3′ (PH20 ΔC491 template)
	Reverse	5′- cacattccacagccaagcttatcgatgataagctgtcaaac -3′ (PH20 ΔC491 template)
D94S	Forward	5′ - caatattttatgttTCTagacttgg -3′
	Reverse	5′ - gtagccaagtctAGAaacata -3′
D103T	Forward	5′ - gctactatccttacataACTtcaatc -3′
	Reverse	5′ - gttactcctgtgattgaAGTtatgta -3′
E149A	Forward	5′ - ttgactgggaaGCAtggagac-3′
	Reverse	5′ - caagtgggtctTGCttcccag -3′

Mutated nucleotides are shown in capital letters.
