# Supplementary material for: Substitution of acidic residues near the catalytic Glu131 leads to human HYAL1 activity at neutral pH via charge-charge interactions
Source: PLoS One. 2024 Aug 9;19(8):e0308370. doi: 10.1371/journal.pone.0308370 (PMC11315327; doi:10.1371/journal.pone.0308370)
Supplement: S1 Fig — (PDF) [file pone.0308370.s002.pdf]

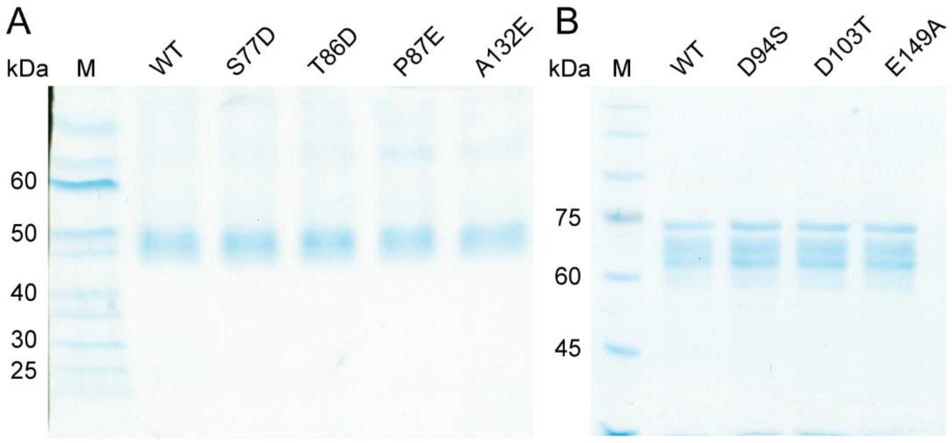

S1 Figure. SDS-PAGE gel after protein purification. (A) HYAL1 WT and single mutants; M: Protein molecular weight marker (Cat. # SM0661, Fermentas). (B) PH20 WT and single mutants; M: Protein molecular weight marker (Cat. # BPM1000, Biomax).
