# Supplementary material for: Substitution of acidic residues near the catalytic Glu131 leads to human HYAL1 activity at neutral pH via charge-charge interactions
Source: PLoS One. 2024 Aug 9;19(8):e0308370. doi: 10.1371/journal.pone.0308370 (PMC11315327; doi:10.1371/journal.pone.0308370)
Supplement: S2 Fig — Structural models of HYAL1 A132H (A) and Cedar bark aphid hyaluronidase (Cinara cedri) (B). (PDF) [file pone.0308370.s003.pdf]

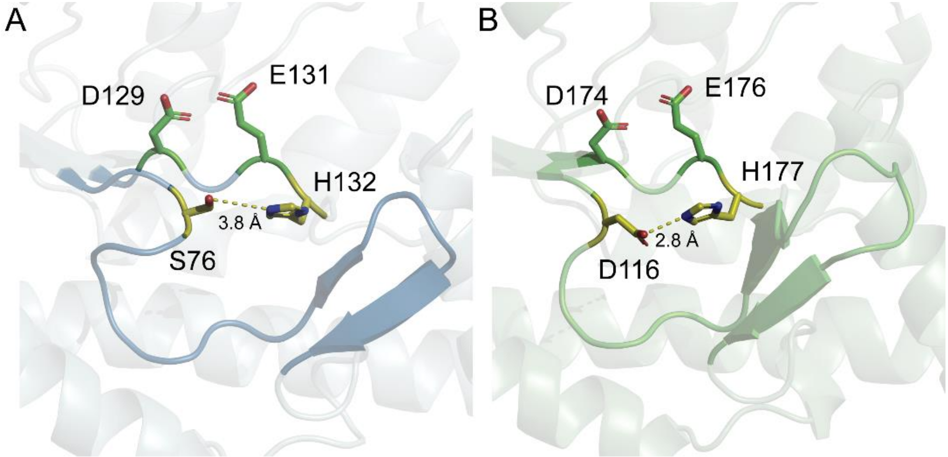

S2 Figure. Structural models of HYAL1 A132H (A) and Cedar bark aphid hyaluronidase (*Cinara cedri*) (B). The two acidic residues in the active site, Asp and Glu, are highlighted in green. In (A), Ser76 and His132 form a hydrogen bond with a distance of 3.8 Å, while in (B), Asp116 and His177 form a salt bridge with a distance of 2.8 Å. The structural models were generated using AlphaFold.
