# Supplementary material for: Substitution of acidic residues near the catalytic Glu131 leads to human HYAL1 activity at neutral pH via charge-charge interactions
Source: PLoS One. 2024 Aug 9;19(8):e0308370. doi: 10.1371/journal.pone.0308370 (PMC11315327; doi:10.1371/journal.pone.0308370)
Supplement: S3 Fig — pH-dependent activity of HYAL1 extended β-hairpin region mutants (A) and Ser76 mutants (B). (PDF) [file pone.0308370.s004.pdf]

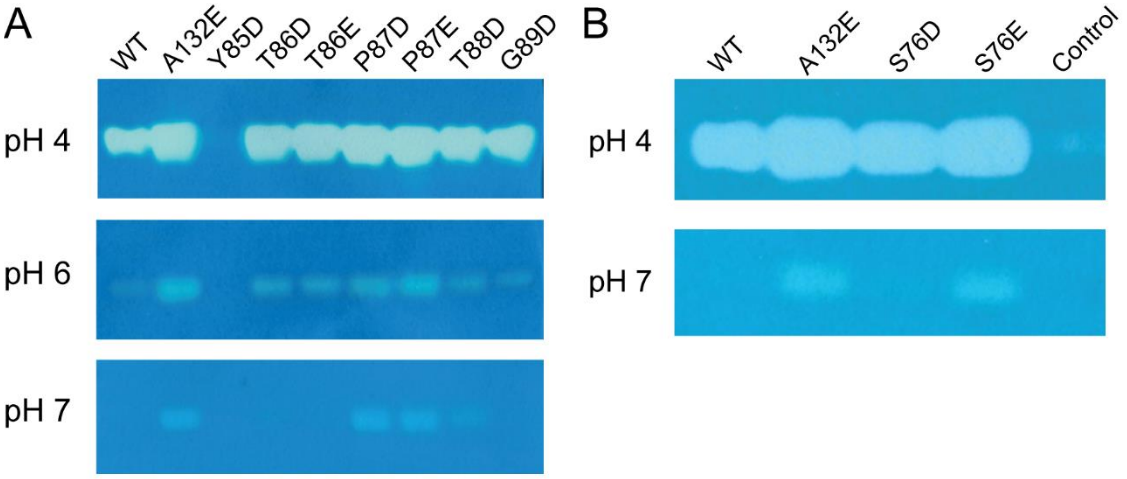

S3 Figure. pH-dependent activity of HYAL1 extended β-hairpin region mutants (A) and Ser76 mutants (B). Additional substitutions in the β-hairpin region include Y85D, P87D, T88D, and G89D (A), while in the β3-loop region are S76D and S76E (B). The acetate buffer at pH 4 comprised 100 mM sodium acetate, 100 mM sodium chloride, and glacial acetic acid. Phosphate buffers at pH 6 and 7 contained 100 mM sodium phosphate and 100 mM sodium chloride.
