# Supplementary material for: Substitution of acidic residues near the catalytic Glu131 leads to human HYAL1 activity at neutral pH via charge-charge interactions
Source: PLoS One. 2024 Aug 9;19(8):e0308370. doi: 10.1371/journal.pone.0308370 (PMC11315327; doi:10.1371/journal.pone.0308370)
Supplement: S4 Fig — Structural models of HYAL1 S76D (A) and S77D (B). (PDF) [file pone.0308370.s005.pdf]

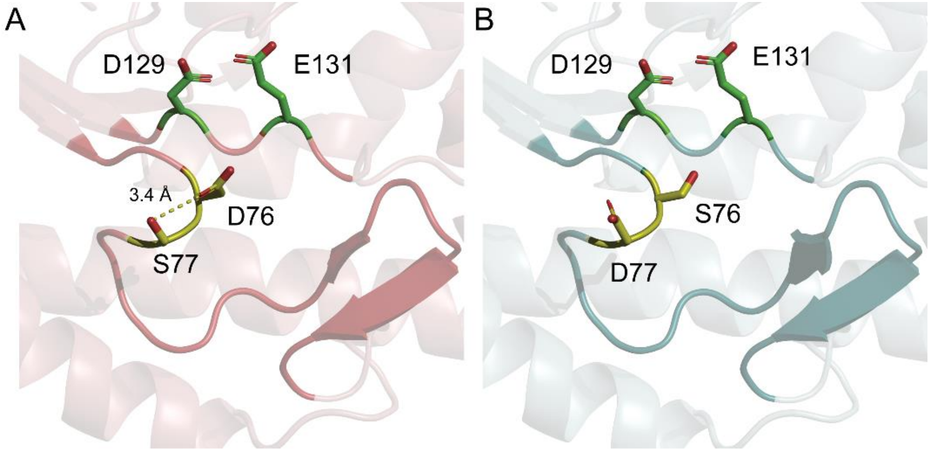

S4 Figure. Structural models of HYAL1 S76D (A) and S77D (B). The acidic residues in the active site, Asp129 and Glu131, are highlighted in green. In (A), Asp76 and Ser77 are depicted in yellow, while in (B), Ser76 and Asp77 are represented in yellow. The structural models were generated using AlphaFold.
