# Supplementary material for: Substitution of acidic residues near the catalytic Glu131 leads to human HYAL1 activity at neutral pH via charge-charge interactions
Source: PLoS One. 2024 Aug 9;19(8):e0308370. doi: 10.1371/journal.pone.0308370 (PMC11315327; doi:10.1371/journal.pone.0308370)
Supplement: S5 Fig — (PDF) [file pone.0308370.s006.pdf]

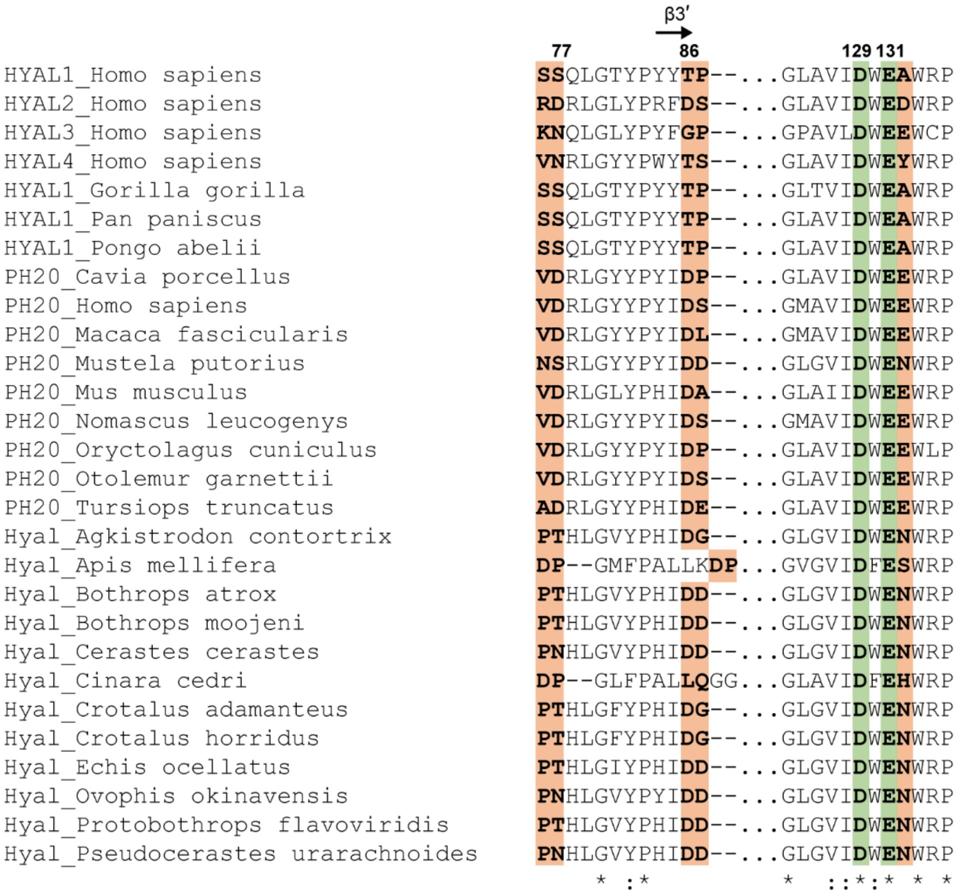

S5 Figure. Sequence comparison of hyaluronidase orthologs. The sequence comparison of hyaluronidases from various species includes: **Human hyaluronidases:** HYAL1 (PDB ID: 2PE4), HYAL2 (UniProt ID: Q12891), HYAL3 (UniProt ID: O43820), HYAL4 (UniProt ID: Q2M3T9), and PH20 (Uniprot ID: P38567). **HYAL1 from other species:** *Gorilla gorilla* (UniProt ID: G3QVZ5), *Pan paniscus* (UniProt ID: A0A2R9BRK8), and *Pongo abelii* (UniProt ID: A0A2J8TGZ1); **PH20 from other species:** *Cavia porcellus* (UniProt ID: P23613), *Macaca fascicularis* (UniProt ID: P38568), *Mustela putorius* (UniProt ID: A0A8U0NMP2), *Mus musculus* (UniProt ID: P48794), *Nomascus leucogenys* (UniProt ID: UPI00122DB420), *Oryctolagus cuniculus* (UniProt ID: P38566), *Otolemur garnettii* (UniProt ID: UPI000643F732), and *Tursiops truncatus* (UniProt ID: A0A2U3UYU4); **Venom hyaluronidases:** *Agkistrodon contortrix* (UniProt ID: JAS04369.1), *Apis mellifera* (PDB ID: 1FCU), *Bothrops atrox* (UniProt ID: AUF71538.1), *Bothrops moojeni* (UniProt ID: ATU85542.1), *Cerastes cerastes* (UniProt ID: A3QVN5), *Cinara cedri* (UniProt ID: VVC33728.1), *Crotalus adamanteus* (UniProt ID: J3S820), *Crotalus horridus* (UniProt ID: T1D6Q3), *Echis ocellatus* (UniProt ID: A3QVN2), *Ovophis okinavensis* (UniProt ID: BAN89413.1), *Protobothrops flavoviridis* (UniProt ID: BAP39986.1), and *Pseudocerastes urarachnoides* (UniProt ID: UMW88234.1).
