# Supplementary material for: Substitution of acidic residues near the catalytic Glu131 leads to human HYAL1 activity at neutral pH via charge-charge interactions
Source: PLoS One. 2024 Aug 9;19(8):e0308370. doi: 10.1371/journal.pone.0308370 (PMC11315327; doi:10.1371/journal.pone.0308370)
Supplement: S6 Fig — (PDF) [file pone.0308370.s007.pdf]

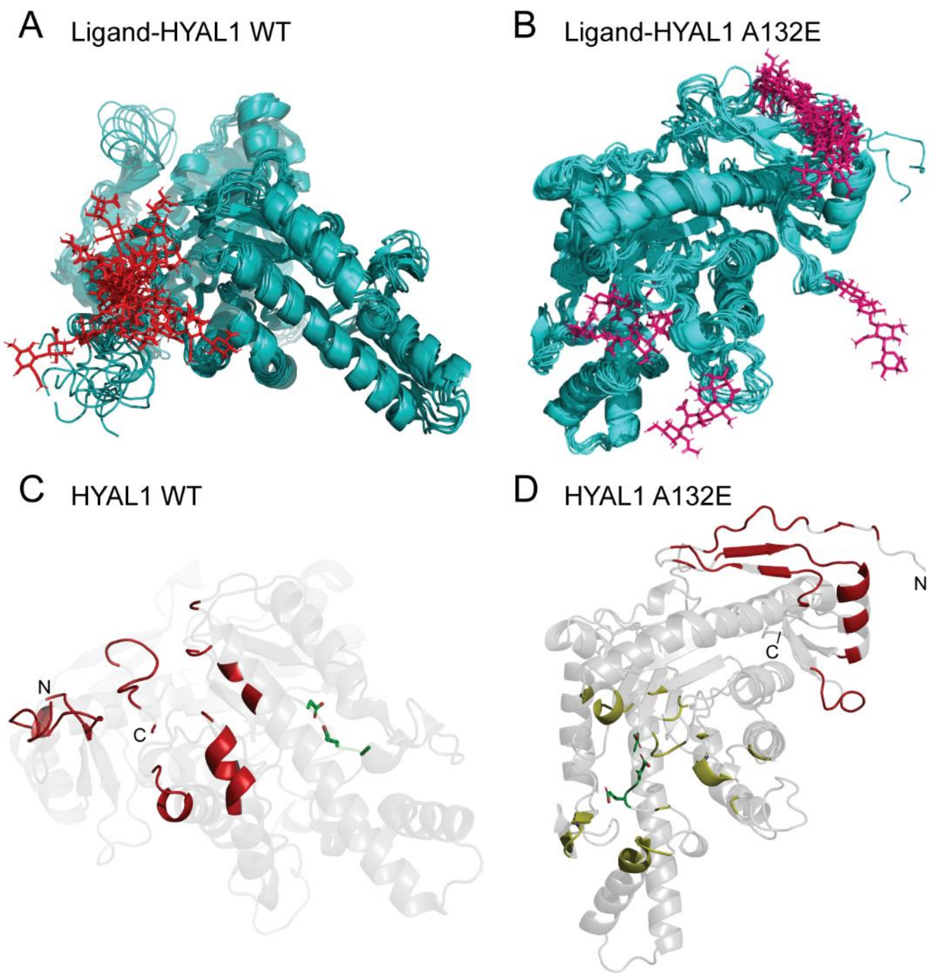

S6 Figure. Docking simulation of HYAL1 WT and A132E with an HA tetrasaccharide. Docking structures of HYAL1 WT (A) and A132E (B) with HA tetrasaccharides. Predicted HA tetrasaccharide-binding sites of HYAL1 WT (C) and A132E (D). In (C) and (D), the ligand-binding sites of each protein located in the N- and C-terminal regions are marked in red. The binding pocket probed by simulation is marked in yellow. Catalytic residues and position 132 are depicted in green.
