# Supplementary material for: Substitution of acidic residues near the catalytic Glu131 leads to human HYAL1 activity at neutral pH via charge-charge interactions
Source: PLoS One. 2024 Aug 9;19(8):e0308370. doi: 10.1371/journal.pone.0308370 (PMC11315327; doi:10.1371/journal.pone.0308370)
Supplement: S7 Fig — (PDF) [file pone.0308370.s008.pdf]

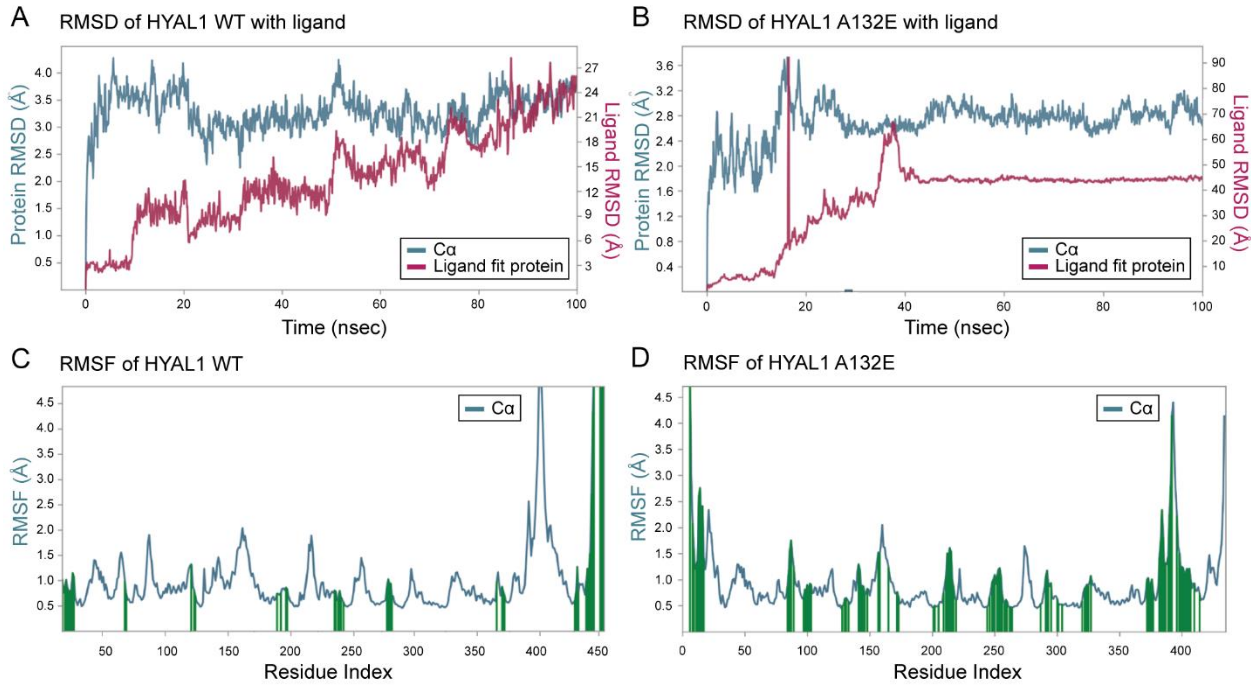

S7 Figure. Molecular dynamics analysis of HYAL1 WT and A132E with an HA tetrasaccharide. (A), (B) RMSD values for HYAL1 WT and A132E and their respective HA tetrasaccharide ligands. (C), (D) RMSF values of HYAL1 WT and A132E upon ligand binding. The green lines indicate the predicted amino acid residues bound to the ligand.
