# Supplementary material for: Substitution of acidic residues near the catalytic Glu131 leads to human HYAL1 activity at neutral pH via charge-charge interactions
Source: PLoS One. 2024 Aug 9;19(8):e0308370. doi: 10.1371/journal.pone.0308370 (PMC11315327; doi:10.1371/journal.pone.0308370)
Supplement: S8 Fig — (PDF) [file pone.0308370.s009.pdf]

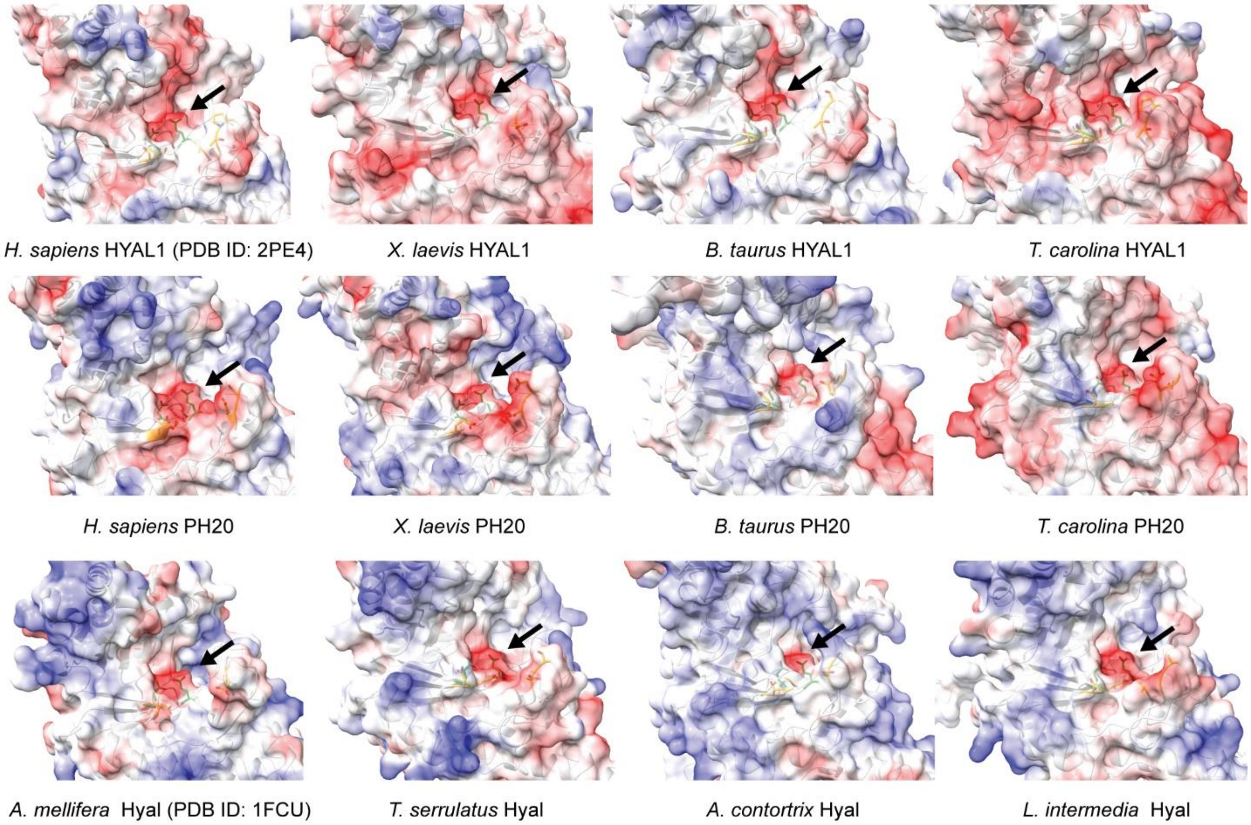

S8 Figure. Surface electrostatic potential of hyaluronidases. The surface electrostatic potential of hyaluronidase structures was generated and analyzed using ChimeraX. Negative, positive, and neutral electrostatic potential regions are indicated by red, blue, and white colors, respectively. The active site of hyaluronidases is marked with a black arrow. Catalytic residues are shown in deep green, while surrounding negatively charged residues are depicted in orange. The following structures were included. HYAL1 structures: *H. sapiens* (PDB ID: 2PE4), *Bos taurus* (UniProt ID: Q5E985), *Terrapene carolina* (UniProt ID: A0A674IY09), and *Xenopus laevis* (UniProt ID: A0A1L8GP88). PH20 structures: *H. sapiens* (UniProt ID: P38567), *B. taurus* (UniProt ID: Q2YDK3), *T. carolina* (UniProt ID: A0A674IDS3), and *X. laevis* (UniProt ID: Q8UVT7). Venom hyaluronidase structures: *Apis mellifera* (PDB ID: 1FCU), *Loxosceles intermedia* (UniProt ID: R4J7Z9), *Agkistrodon contortrix* (NCBI ID: JAS04369.1), and *Tityus serrulatus* (NCBI ID: AHF72517.1). The predicted structures were obtained from UniProt.
