# Supplementary material for: Substitution of acidic residues near the catalytic Glu131 leads to human HYAL1 activity at neutral pH via charge-charge interactions
Source: PLoS One. 2024 Aug 9;19(8):e0308370. doi: 10.1371/journal.pone.0308370 (PMC11315327; doi:10.1371/journal.pone.0308370)
Supplement: S1 Raw images — (PDF) [file pone.0308370.s010.pdf]

S1 Raw images.

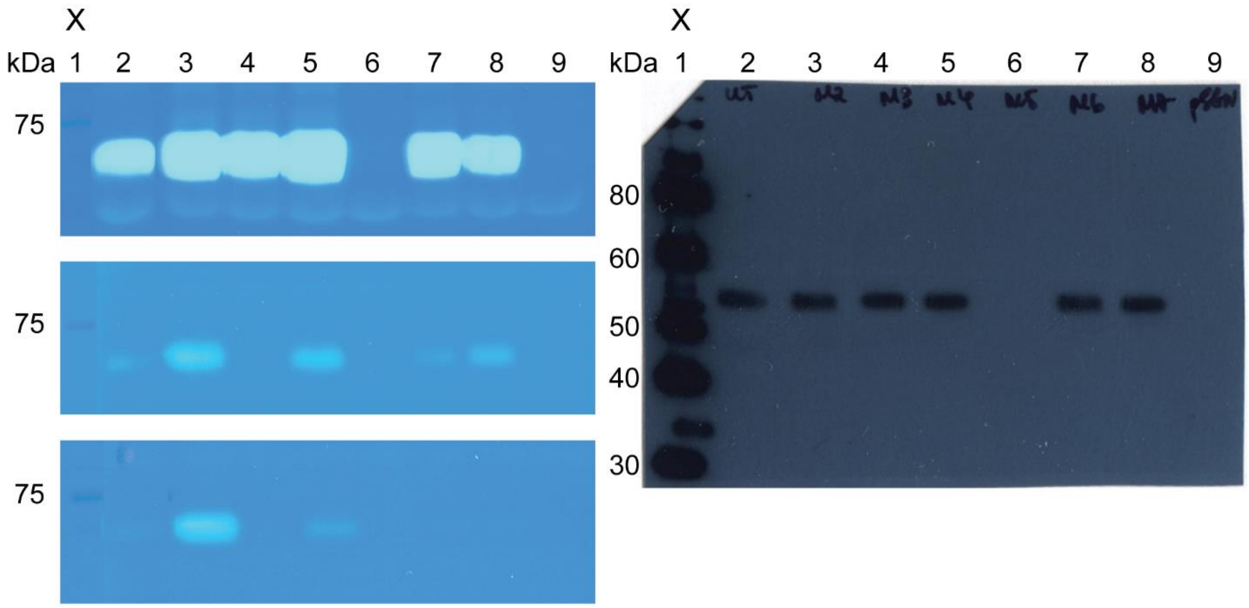

Fig 2A. Substrate gel assay results at pH 4, 6, and 7 and Western blot analysis for HYAL1 WT and Ala132 mutants. Protein molecular weight marker (Cat. # BPM1000, Biomax) and Western marker (Cat. # WM1000, YesBlot).

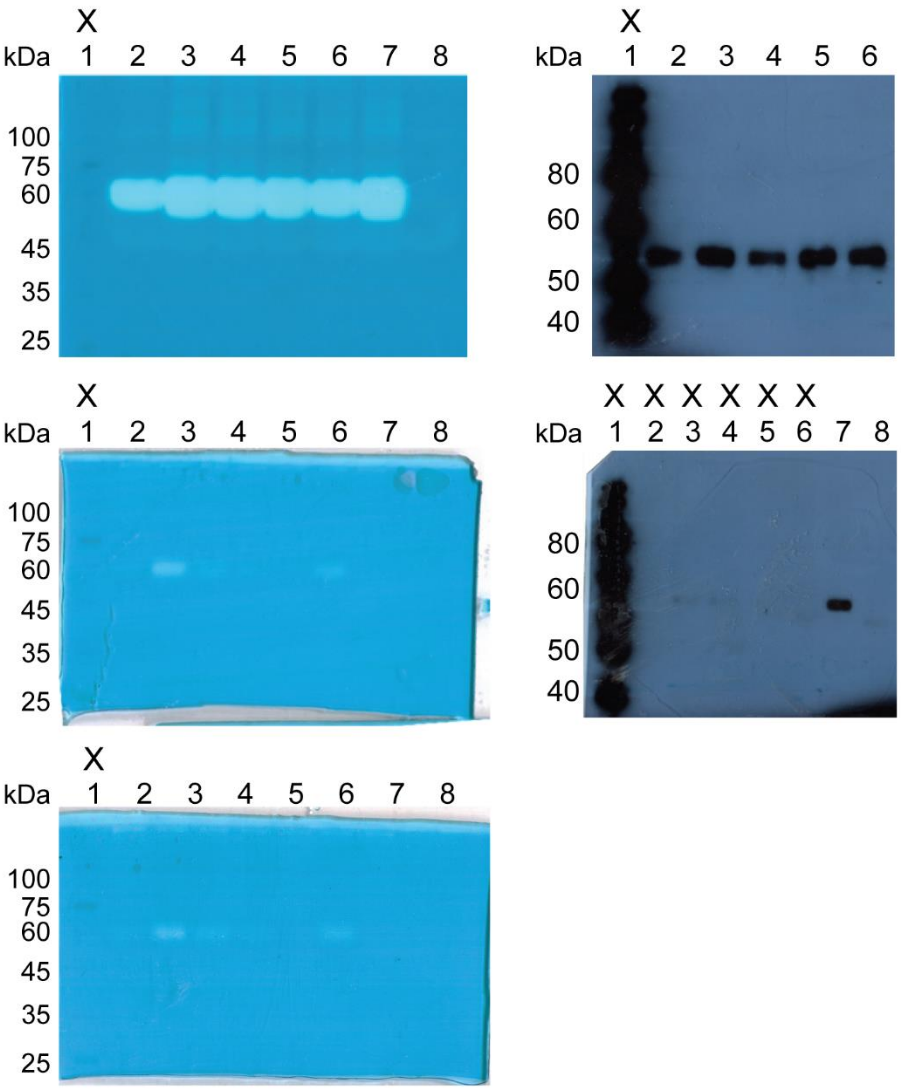

Fig 3A. Substrate gel assay results at pH 4, 6, and 7 and Western blot analysis for HYAL1 WT and β3-loop and β-hairpin mutants. Protein molecular weight marker (Cat. # BPM1000, Biomax) and Western marker (Cat. # WM1000, YesBlot).

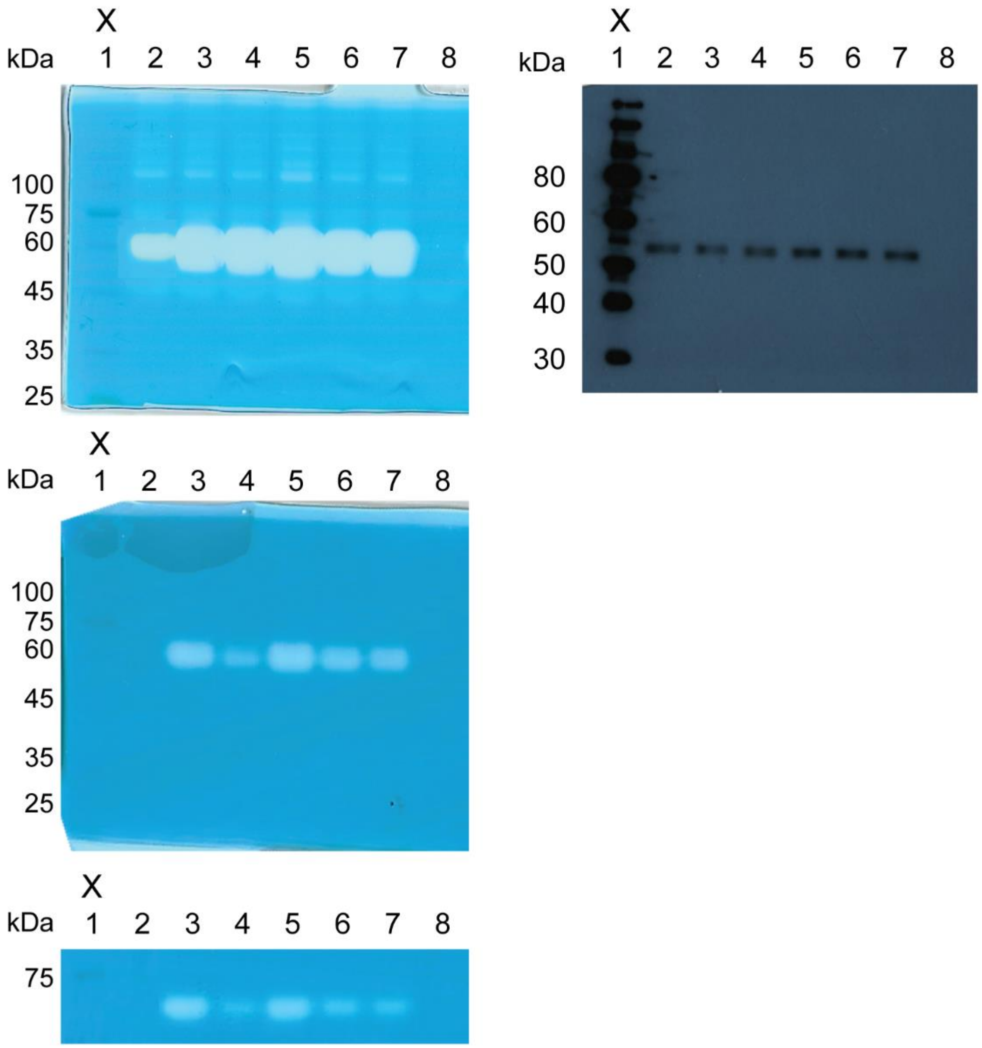

Fig 4A. Substrate gel assay results at pH 4, 6, and 7 and Western blot analysis for HYAL1 WT and β3-loop and β-hairpin mutants with double substitutions. Protein molecular weight marker (Cat. # BPM1000, Biomax) and Western marker (Cat. # WM1000, YesBlot).

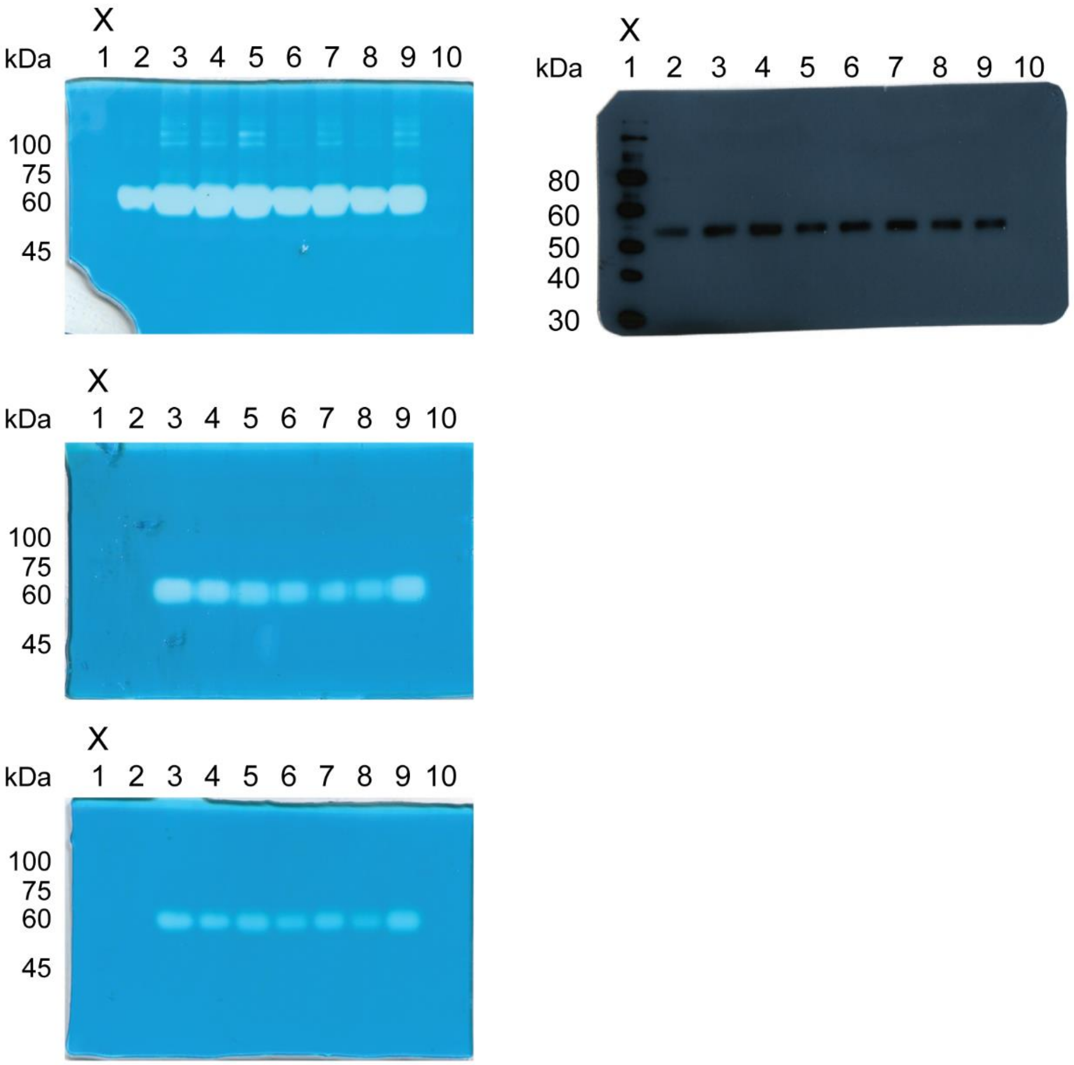

Fig 5A. Substrate gel assay results at pH 4, 6, and 7 and Western blot analysis for HYAL1 WT and double, triple substitutions in the β3-loop, β-hairpin, and Ala132 mutants. Protein molecular weight marker (Cat. # BPM1000, Biomax) and Western marker (Cat. # WM1000, YesBlot).

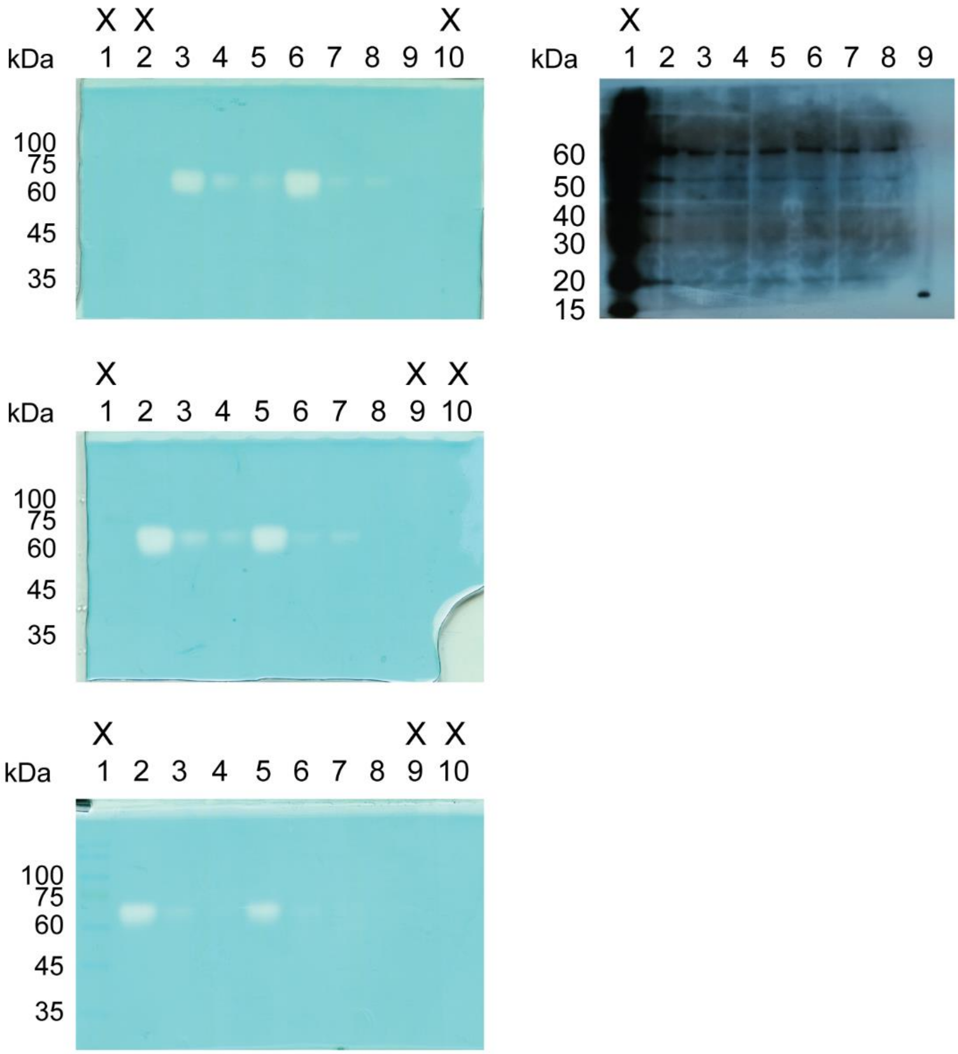

Fig 6A. Substrate gel assay results at pH 4, 6, and 7 and Western blot analysis for PH20 WT and the β3-loop, β-hairpin, and Glu149 mutants. Protein molecular weight marker (Cat. # BPM1000, Biomax) and Western marker (Cat. # WM1000, YesBlot).
